# Quantification of Plant Root Species Composition in Peatlands Using FTIR Spectroscopy

**DOI:** 10.3389/fpls.2020.00597

**Published:** 2020-05-19

**Authors:** Petra Straková, Tuula Larmola, Javier Andrés, Noora Ilola, Piia Launiainen, Keith Edwards, Kari Minkkinen, Raija Laiho

**Affiliations:** ^1^Natural Resources Institute Finland (LUKE), Helsinki, Finland; ^2^Department of Forest Sciences, University of Helsinki, Helsinki, Finland; ^3^Department of Agricultural Sciences, University of Helsinki, Helsinki, Finland; ^4^Financial and Administrative Services, Education Department, City of Vantaa, Vantaa, Finland; ^5^Department of Ecosystem Biology, University of South Bohemia, České Budějovice, Czechia

**Keywords:** FTIR, calibration model, dead roots, fine roots, peatland, plant root composition, plant functional type (PFT), root chemistry

## Abstract

Evidence of plant root biomass and production in peatlands at the level of species or plant functional type (PFT) is needed for defining ecosystem functioning and predicting its future development. However, such data are limited due to methodological difficulties and the toilsomeness of separating roots from peat. We developed Fourier transform infrared (FTIR) spectroscopy based calibration models for quantifying the mass proportions of several common peatland species, and alternatively, the PFTs that these species represented, in composite root samples. We further tested whether woody roots could be classified into diameter classes, and whether dead and living roots could be separated. We aimed to solve whether general models applicable in different studies can be developed, and what would be the best way to build such models. FTIR spectra were measured from dried and powdered roots: both “pure roots”, original samples of 25 species collected in the field, and “root mixtures”, artificial composite samples prepared by mixing known amounts of pure roots of different species. Partial least squares regression was used to build the calibration models. The general applicability of the models was tested using roots collected in different sites or times. Our main finding is that pure roots can replace complex mixtures as calibration data. Using pure roots, we constructed generally applicable models for quantification of roots of the main PFTs of northern peatlands. The models provided accurate estimates even for far distant sites, with root mean square error (RMSE) 1.4–6.6% for graminoids, forbs and ferns. For shrubs and trees the estimates were less accurate due to higher within-species heterogeneity, partly related to variation in root diameter. Still, we obtained RMSE 3.9–10.8% for total woody roots, but up to 20.1% for different woody-root types. Species-level and dead-root models performed well within the calibration dataset but provided unacceptable estimates for independent samples, limiting their routine application in field conditions. Our PFT-level models can be applied on roots separated from soil for biomass determination or from ingrowth cores for estimating root production. We present possibilities for further development of species-level or dead-root models using the pure-root approach.

## Introduction

Root-mediated carbon (C) fluxes represent the major information gap when estimating C stocks and C transformations of any ecosystem that supports plant communities. Plants are the main drivers of the whole ecosystem productivity, and plant roots, “the hidden part” of the plant community, may comprise an equal or even greater part of the biomass or the annual biomass production compared to “the obvious part” aboveground (Persson, 1983; Jackson et al., 1996; Gower et al., 2001). Remains from roots and root-associated microorganisms then may form 50–70% of sequestered soil C (Clemmensen et al., 2013). Still, root systems in most plant communities are poorly understood.

In peatlands, the wet C hotspots of our planet (e.g., Page et al., 2011; Nichols and Peteet, 2019), root studies are especially rare (e.g., Iversen et al., 2018). Yet, root production is likely a major C flux in sites that are characterized by graminoids, such as sedge fens (Saarinen, 1996), and sites with a tree stand (Bhuiyan et al., 2017; Minkkinen et al., 2018), especially when there is an abundant shrub understory (Finér and Laine, 1998). Naturally forested sites are quite common in many peatland regions (Rydin and Jeglum, 2013). There are further nearly 150 000 km^2^ of peatlands drained for forestry purposes, mostly in northern Europe (Paavilainen and Päivänen, 1995; Joosten and Clarke, 2002), where the vascular plant composition is shrub and tree dominated (Laine et al., 1995; Laiho et al., 2003).

Graminoids are adapted to produce large root biomass even in waterlogged conditions and very deep anoxic soil layers (Bernard et al., 1988; Saarinen, 1996; Proctor and He, 2019), compared to which shrubs or trees require drier soil conditions and are thus more shallow-rooting, but still producing significant root mass (Finér and Laine, 2000; Iversen et al., 2018). In addition to producing C inputs into the soil as root litter and root exudates, active plant roots may affect ecosystem functioning by, e.g., shaping the soil microbial community and its functions (e.g., Robroek et al., 2015). Such effects typically depend on plant species or plant functional type (PFT) (Straková et al., 2011; Peltoniemi et al., 2012; Robroek et al., 2015; Kaštovská et al., 2018), even though studies on specific root impacts are still rare. Changes in root biomass, rooting depth and/or species/PFT composition as a response to environmental or global changes may therefore strongly influence responses of the whole ecosystem. The lack of root-related data shows up as high uncertainty in ecosystem models predicting current and future functioning of peatlands (Frolking et al., 2010, 2011), and uncertainty in soil organic C monitoring in general (Lorenz and Lal, 2010; Jandl et al., 2014).

The shortage of knowledge on root-mediated C fluxes is mainly due to prevailing methodological difficulties. Root production may be related to the above-ground vegetation characteristics (Murphy et al., 2009a, b; Murphy and Moore, 2010), but unfortunately, general models do not exist and thus direct root measurements are still needed. Separating roots from soil and live roots from dead roots is extremely laborious; especially so when it comes to peat soils that consist of plant remains, including roots, at various stages of decay (Sjörs, 1991). The same holds for species identification, which is typically carried out by hand-sorting and visual classification using morphological criteria and anatomical microscopic inspections (e.g., Bhuiyan et al., 2017). Such identification is time consuming, and subjective even for personnel with high level of expertise, and thus largely constrained in mixed stands or species-rich systems. Consequently, considerable effort has lately been invested to developing methods for root species identification and quantification, including DNA-based techniques (e.g., Mommer et al., 2011), pyrolysis (White et al., 2011) or plant wax markers (Dawson et al., 2000; Roumet et al., 2006). Each of these methods has its own limitations, commonly high cost, which largely reduces the number of samples that can be analyzed. Spectroscopy methods, near infrared (NIR) and recently Fourier transform infrared (FTIR), have been identified as the most promising (review by Rewald and Meinen, 2013, and references therein).

Near infrared and FTIR spectroscopy are non-destructive physical methods. The spectrum of light absorbed by a sample in the near-infrared (1100–2500 nm) or infrared (4000–400 cm^–1^) region gives a chemical signature of the sample, providing information about the presence, character and abundance of chemical bonds or functional groups. Generally, infrared spectroscopy provides such advantages as the possibility to analyze large sample sets in relatively short time at low cost, with little sample preparation needed and no chemicals used. In addition, FTIR-ATR technique requires only small amounts of sample material, which is in many cases limited in root studies. Chemometrics then enable quantification of the required component (e.g., percentage of one plant species in the multispecies root mixture) using multivariate calibration of the spectroscopy data (Esbensen et al., 2002). The process of calibration is similar for both NIR and FTIR, with FTIR shown to be somewhat more precise (Bellon-Maurel and McBratney, 2011) and in principle enabling also direct interpretation of the spectral properties.

For root species identification or quantification, NIR or FTIR spectroscopy has already been applied on agricultural crop and weed species (Rumbaugh et al., 1988; Roumet et al., 2006; Picon-Cochard et al., 2009; Naumann et al., 2010; Kusumo et al., 2011; White et al., 2011; Meinen and Rauber, 2015; Legner et al., 2018; Streit et al., 2019) and forest tree species (Lei and Bauhus, 2010; Domisch et al., 2015; Tong et al., 2016; Finér et al., 2017), and to separate live roots from the dead for forage species (Picon-Cochard et al., 2009). The number of plant species contained in the root mixtures has ranged from two to five. These studies have shown the potential of NIR/FTIR to relate roots to plant species in specific studies using local root materials.

To our knowledge, NIR or FTIR spectroscopy has not been used by others to determine the root composition of peatland plant species. Neither have there been attempts to create identification methods for PFTs instead of individual species, even though the PFT-level might be sufficient and in some cases more applicable in studies covering a wide range of conditions and species. Also, in previous studies, it was not tested whether the models developed for their specific purposes could be applicable more generally for the species studied. Preparing root mixtures for calibration data for each study site specifically is the most laborious part of utilizing the spectroscopy methods.

We aimed to develop FTIR-based calibration models for predicting the mass proportions of (i) several common peatland species, and alternatively, (ii) the PFTs that these species represented, in composite root samples. We further tested whether (iii) roots of woody plants could be classified into different diameter classes with such models, and (iv) the possibility to estimate proportions of dead and living roots with 4 plant species. Furthermore, we tested (v) whether pure roots (single-species and/or single diameter samples) could replace complex mixtures as calibration data. We strove for general applicability of the models for different peatland sites and samples that would represent, e.g., roots separated from soil samples for biomass determination or roots separated from ingrowth cores for estimating root production. We hypothesized that in root composite samples:

**(H1):** Roots of all peatland plant species or PFTs can be distinguished (principal component analysis, cluster analysis) and quantified (partial least squares regression calibration models) using FTIR, assuming that the between-species (interspecific) chemical variation captured by FTIR is higher than the within-species (intraspecific) heterogeneity.

**(H2):** Proportions of very fine (diameter ≤ 0.5 mm), fine (diameter < 2 mm) and coarser (diameter 2–10 mm) roots of shrubs and trees can be distinguished and quantified using FTIR, assuming that roots of the defined diameter classes have different FTIR signatures but the differences do not override between-species chemical variation.

**(H3):** Proportions of dead and living roots can be quantified using FTIR, assuming that dead and living roots have different FTIR signatures but the differences do not override between-species chemical variation.

## Materials and Methods

### The Root Materials

**FIGURE 1:**
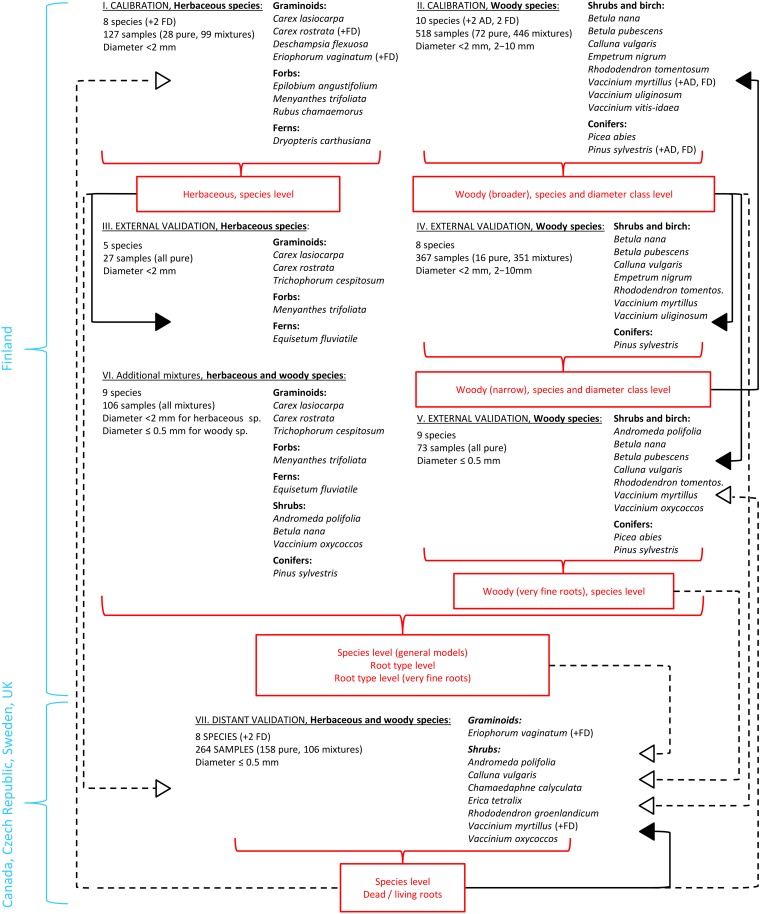
Overview on the utilization of different root materials in calibration and validation of the models. Roman numerals refer to the sample sets as in Table 1. Full arrows represent external validation, empty arrows with dash lines represent distant validation. Abbreviations for dead roots: AD, artificially dead; FD, field-dead.

We gathered an extensive set of plant roots for building FTIR based calibration models and their validation. The roots were altogether of 25 plant species and included both herbaceous (graminoids, forbs and ferns) and woody (shrubs, a broadleaf tree, coniferous trees) plants (Table 1). The samples were organized as several specific sample sets used for calibration and validation as outlined in Table 1 and Figure 1. Since we aimed to create generally applicable calibration models, we included naturally occurring variation in root chemistry extensively in the calibration sample sets. To include within-site variation, roots of each species were collected from several plants at different locations of each site. To include between-site variation, roots were collected from several study sites for each species. Only the roots of graminoid *Deschampsia flexuosa* and forb *Epilobium angustifolium* in the *calibration sample set I*, which are not typical peatland species but still may appear in disturbed drained peatlands, were collected at one study site only. The sites varied in soil type, water-level regime, nutrient regime, and climatic conditions. The roots were collected at different times of the growing season from May to October, in different years. Collection is described in the Supplementary Material.

**TABLE 1:** Plant species and frequency of their occurrence in different subsets of the root samples, with number of pure root substrates in parentheses.

			**Calibration sets:**		**External validation sets:**				**Distant validation sets:**			
					
			**I**	**II**	**III**	**IV**	**V**	**VI**	**VII**	**VII**	**VII**	**VII**
			**FI**	**FI**	**FI**	**FI**	**FI**	**FI**	**CA**	**CZ**	**SE**	**UK**
**Species**	**Species abbreviation**	**Number of sites**	***n* = 127 (28)**	***n* = 518 (72)**	***n* = 27 (27)**	***n* = 367 (16)**	***n* = 73 (73)**	***n* = 106**	***n* = 57 (21)**	***n* = 90 (90)**	***n* = 44 (8)**	***n* = 73 (39)**
**Herbaceous plants:**												
*Graminoids:*												
*Carex lasiocarpa*	CL	2	29 (4)		9 (9)			37				
*Carex rostrata*	CR	2	35 (4)		6 (6)			39				
*Carex rostrata* FD		2	8 (2)									
*Deschampsia flexuosa*	DF	1	23 (1)									
*Eriophorum vaginatum*	EV	8	40 (4)						22 (4)	5 (5)	23 (5)	17 (1)
*Eriophorum vaginatum* FD		5	11 (4)							30 (30)		
*Trichophorum cespitosum*	TC	2			4 (4)			38				
*Forbs:*												
*Epilobium angustifolium*	EA	1	23 (1)									
*Menyanthes trifoliata*	MT	2	28 (2)		4 (4)			31				
*Rubus chamaemorus*	RC	3	28 (4)									
*Ferns:*												
*Dryopteris carthusiana*	DC	2	25 (2)									
*Equisetum fluviatile*	EF	2			4 (4)			26				
**Woody plants:**												
*Shrubs and broadleaf trees:*												
*Andromeda polifolia*	AP	3					11 (11)	50			38 (2)	
*Betula nana*	BN	3		115 (6)		147 (2)	9 (9)	42				
*Betula pubescens*	BP	2		124 (4)		139 (2)	6 (6)					
*Calluna vulgaris*	CV	3		99 (3)		155 (2)	4 (4)					58 (20)
*Chamaedaphne calyculata*	CC	1							42 (6)			
*Empetrum nigrum*	EN	2		124 (3)		150 (2)						
*Erica tetralix*	ET	1										52 (18)
*Rhododendron groenlandicum*	RG	1							44 (8)			
*Rhododendron tomentosum*	RT	2		131 (6)		116 (2)	6 (6)					
*Vaccinium myrtillus*	VM	5		160 (10)		152 (2)	13 (13)			28 (28)		
*Vaccinium myrtillus* FD, AD		3		16 (2)						27 (27)		
*Vaccinium oxycoccos*	VO	3					10 (10)	44	3 (3)		45 (1)	
*Vaccinium uliginosum*	VU	3		122 (4)		147 (2)						
*Vaccinium vitis-idaea*	VV	3		87 (2)								
*Coniferous trees:*												
*Picea abies*	PA	5		159 (7)			8 (8)					
*Pinus sylvestris*	PS	7		233 (21)		150 (2)	6 (6)	32				
*Pinus sylvestris* FD, AD		4		62 (4)								

*Roman numerals refer to the sample sets as in Figure 1. Dead roots abbreviations: AD, artificially dead roots; FD, field-dead roots. Sample origins: CA, Canada; CZ, Czechia; SE, Sweden; UK, United Kingdom.*

Our initial focus was on fine roots that we defined as roots of diameter < 2 mm for all plant species. Coarser woody roots of diameter 2–10 mm were included into the datasets as substantial part of the belowground biomass of woody plants is formed by this fraction (e.g., Murphy et al., 2009b; Weishampel et al., 2009), and they are not covered by allometric equations used for estimating coarse root biomass of woody species (e.g., Laiho and Finér, 1996). However, when we started to apply the modified ingrowth core method (Laiho et al., 2014) for estimating root production, the results revealed that 92% of the ingrown roots in peatland forests within 3 year incubation were of diameter ≤ 1 mm (Bhuiyan et al., 2017), of which the majority was as thin as ≤ 0.5 mm. Thus a separate class of very fine roots of diameter ≤ 0.5 mm was furthermore added for woody plants.

The study included two types of dead roots, field-dead roots that were collected from living plants (*Carex rostrata*, *Eriophorum vaginatum*, *Pinus sylvestris* and *Vaccinium myrtillus*) in the field and separated from living roots using morphological criteria (color, structure and strength), as well as artificially dead roots of *Pinus sylvestris* and *Vaccinium myrtillus* produced in a root mortality treatment where root death was induced by desiccation. The treatment is described in the Supplementary Material. The aim of the artificial killing was to obtain dead, but still largely undecomposed roots.

### Root Mixtures

Dried root samples were powdered with an oscillating ball-mill. Root mixtures containing known mass proportions of roots of different plant species (and diameter class for woody roots) were prepared by weighing and mixing pure root powders within the given sample set (Table 1 and Figure 1), similarly as in, e.g., Lei and Bauhus (2010), Domisch et al. (2015), Meinen and Rauber (2015), Tong et al. (2016) and Finér et al. (2017). The proportion of each plant species in the mixtures ranged from 0 to 100%, on a dry mass basis. For calibration and external validation purposes root mixtures were prepared with 2–7 species components, the different plant species thus had from tens to hundreds occurrences in the mixtures (Table 1). For distant validation purposes root mixtures were prepared with 2–3 species components. Altogether we utilized about 1500 samples.

### FTIR Measurements

Measurements were done on both the “pure roots,” original samples of each species and diameter class available, and the “root mixtures” prepared. FTIR spectra of all samples, except for roots from the sites in Canada, Sweden and United Kingdom in the *distant validation sample set VII*, were obtained with a Bruker VERTEX 70 FTIR spectrometer (Bruker Optics, Germany) with a horizontal diamond ATR sampling accessory. Pulverized samples were placed directly on the diamond crystal (diameter 1.8 mm) and a MIRacle high-pressure digital clamp was used to achieve even distribution and contact of the sample and crystal. Each spectrum consisted of 65 averaged absorbance measurements between 4000 and 650 cm^–1^, with 2 cm^–1^ resolution. Opus software was used to collect the measured data.

For the remaining samples, FTIR spectra were obtained with Shimadzu IRPrestige-21 FTIR spectrometer (Shimadzu Corporation, Japan) with a horizontal diamond ATR sampling accessory and the same measurement settings as described above for the Bruker spectrometer. IRsolution software was used to collect the measured data. Compatibility of spectra from the two different instruments was ensured by measuring several standard samples with both instruments. The only adjustment needed before merging the data was interpolation of the FTIR spectra measured with the Shimadzu spectrometer to the exact wavenumber range of the Bruker spectrometer. This was done using the Unscrambler software.

### Multivariate Data Analyses

FTIR data were smoothed (Savitzky-Golay smoothing with second polynomial order and 11 smoothing points), baseline corrected, mean normalized and transformed by second derivative (Savitzky-Golay derivative with second polynomial order and 15 smoothing points). This combination of pre-treatments was selected after testing different pre-treatments, including first derivative, standard normal variate (SNV), de-trending, multiplicative scatter correction (MSC), attenuated total reflectance (ATR) correction (Esbensen et al., 2002). The spectral parts 4000–2978, 2828–1752 and 772–650 cm^–1^ were excluded from further analyses due to lack of relevant information in these regions after the transformation. FTIR spectra used in the analyses thus consisted of transformed absorbance values at 2980-2830 and 1750-770 cm^–1^, with 2 cm^–1^ resolution.

Cluster analysis, with Ward’s algorithm and Euclidian distance, and principal component analysis (PCA) were used to compare the roots and define their grouping into chemically distinct root types using the FTIR data of pure (non-mixed) samples as the independent variables (X-matrix).

Partial least squares regression (PLSR) was used to build the calibration models, with known percentage of each plant species (and/or diameter class, root type, or dead root variant) in composite samples as the dependent variable (Y) and FTIR data as the independent variables (X-matrix). Three methods were used for the model validation: (1) internal leave-one-out cross validation (Esbensen et al., 2002), (2) external validation by using newly collected local (Finland) samples and (3) distant validation by using newly collected distant (Canada, Czechia, Sweden, United Kingdom) samples. Further notes on the validation are provided in Supplementary Material.

For both the calibration models and their validations, the values of the dependent variable predicted as less than 0% were set to 0% while those predicted as greater than 100% were set to 100%. Overall, this did not affect the results considerably. However, in cases where there were no roots of the predicted species or root type in the composite samples and the estimates were all negative (which is in fact a very good outcome), this resulted in root mean square error (RMSE) of the estimates equal to 0 indicating perfect fit of the model to the data, almost never achieved in practise. The *r*^2^ values, slope and offset of the regression line, root mean square error (RMSE) of calibration and validation were used to evaluate the models. A good calibration model should have high *r*^2^ value, slope close to 1, offset close to 0, low RMSE of both calibration and validation, and a relatively low number of factors (PCs) in order to avoid inclusion of signal noise in the models. For evaluating the reliability of the predictions on new samples, sample deviation and inliner statistic (minimum Mahalanobis distance to the calibration samples) against Hotelling’s *T*^2^ statistic were used (Esbensen et al., 2002).

The Unscrambler 10.3 (Camo Process AS; Oslo, Norway) and Canoco 5 (Microcomputer Power, United States) software packages were used for the data analyses.

### The Progress for Developing Different Sets of Models and Their Validation

Our data set allowed us to test and compare different ways of constructing the models. Unlike in previous studies, we were also able to validate the models on fully independent local (Finland; external validation) and distant (Canada, Czechia, Sweden, United Kingdom; distant validation) samples.

#### Grouping Roots of Different Species Into More Universal Root Types: PCA and Cluster Analysis (H1, H2)

We started by PCA and cluster analysis on pure root samples from Finland, the *calibration* and *external validation sample sets I–V* (Table 1 and Figure 1), based on which we defined grouping of roots into different root types. The root types should provide more universal grouping of roots than species level, and generally represented different PFTs: graminoids, forbs, ferns, shrubs and trees, as well as different diameter classes for woody roots: very fine (diameter ≤ 0.5 mm), fine (diameter < 2 mm) and coarser (diameter 2–10 mm).

The PCA and cluster analysis were validated both externally (Finland) and distantly (Canada, Czechia, Sweden and United Kingdom). This was done by projection of the *distant validation sample set VII* into the PCA defined by the *calibration* and *external validation sample sets I–V*, or using them all together in a new cluster analysis. External validation was also done by re-constructing the PCA and cluster analysis for only subset of the Finnish samples (*calibration sample sets I, II*) and then using the other subset, collected in different years (*external validation sample sets III–V*) for the external validation (not shown).

External and distant validation of the outcomes ensured not only a correct grouping of roots of several species and/or diameters into more universal root types, but also tested the possibility to use PCA or cluster analysis as a simple tool for rough identification of newly collected root samples.

#### PLSR Calibration Models: Mixtures and Pure Root Approach

We continued by constructing PLSR calibration models for quantification of the mass proportions of the defined root types or individual species. We first used the approach of constructing the calibration models by creating artificial mixtures with varying proportions of the different plant species or root types in the mixtures, similarly to the earlier studies (Rumbaugh et al., 1988; Roumet et al., 2006; Picon-Cochard et al., 2009; Lei and Bauhus, 2010; Naumann et al., 2010; Kusumo et al., 2011; White et al., 2011; Domisch et al., 2015; Meinen and Rauber, 2015; Tong et al., 2016; Finér et al., 2017; Legner et al., 2018; Streit et al., 2019). Then we invented an alternative approach of constructing the calibration models using only pure root substrates (single species and/or diameter class), thus skipping the need of creating the artificial mixtures and having only 0% and 100% values on the calibration curve.

##### PLSR calibration models at the level of root type

Calibration models at the level of root type were constructed using all available roots samples from Finland, the *calibration* and *external validation sample sets I–VI.* These models thus included woody roots of different diameter classes (≤ 0.5 mm, < 2 mm, 2–10 mm) and we made attempts to quantify their proportions for woody plants in composite samples. Another set of root type level models was constructed using only the *calibration* and *external validation sample sets I, III, V and VI.* These calibration models thus included only the very fine (diameter ≤ 0.5 mm) roots of woody plants, while keeping all the herbaceous plant roots.

Additionally, both of these model sets were also constructed using only the pure roots (no mixtures) of the specified sample sets.

##### PLSR calibration models at the level of plant species and diameter class (H1, H2)

Calibration models at the level of plant species were first constructed separately for herbaceous (*calibration sample set I*) and woody (*calibration* and *external validation sample sets II, IV, V*) plants. The separation of herbaceous and woody plants reduced the number of species included in the models, and allowed more detailed testing and investigation of the models when they were not affected by the presence of the other plant type. As the herbaceous and woody plants coexist in real peatland sites, however, they were in the end merged in the general species level calibration models (*calibration* and *external validation sample sets I*–*VI*). This allowed us to test if the model performance is negatively affected by the presence of both herbaceous and woody plants and the increased number of species in the models.

Next, we again constructed alternative calibration models using pure roots of selected herbaceous and woody plants. Three species (*Eriophorum vaginatum*, *Andromeda polifolia*, *Vaccinium oxycoccos*) of the *calibration* and *external validation sample sets I* and *V* were used. These models were compared with the mixtures models constructed for the same species using samples from the site in Sweden in the *distant validation sample set VII*. We further explored the possibility to apply this pure root approach on increased number of species. We used pure roots of 16 species (graminoids, forbs, ferns, shrubs and trees; *calibration* and *external validation sample sets I* and *IV*) and constructed pure root models for each of the species. Then we used artificial mixtures containing known mass proportions of roots of the given species (prepared from the pure roots used for the calibration) for the model validation, which allowed us to test whether there is any interference between the individual pure root components in the mixture spectra.

For woody plants, our data set allowed us to perform detailed testing of models that distinguish both species and diameter class. We started by “narrow” calibration models at the level of plant species as well as diameter class (diameter < 2 mm and 2–10 mm); narrow in the sense that only one study site and pooled samples for each species and diameter class were included in the calibrations (*external validation sample set IV*). Such narrow models were presented in most earlier root studies (e.g., Roumet et al., 2006; Picon-Cochard et al., 2009; Meinen and Rauber, 2015; Tong et al., 2016) and if such models worked outside the calibration sample set, they would offer a rather fast and simple way of root quantification using a limited number of samples.

Then, “broader” calibration models were prepared so that for each species roots of several different study sites collected at several sampling times were used. Thus, we increased the variation covered by the samples (*calibration sample set II*). Diameters < 2 mm and 2–10 mm were in this case pooled for each species, site and sampling time in known proportions.

Additionally, we constructed species level calibration models focusing on very fine roots (diameter ≤ 0.5 mm, *external validation sample set V*). Due to the very limited amount of available root material of the very fine diameter, root mixtures were not prepared and the models were constructed including only a limited number of pure root samples.

##### Dead roots (H3)

We first made attempts to construct calibration models for quantification of dead roots of four plant species in composite samples with other species. We used dead roots of *Carex rostrata*, *Eriophorum vaginatum, Pinus sylvestris* and *Vaccinium myrtillus* in mixtures with other plant species within the *calibration datasets I* and *II* (Table 1 and Figure 1). This approach did not work well due to insufficient root material for creating robust calibration models and their validation (results not shown).

So we selected a different approach and explored the possibilities and limits of dead roots quantification using calibration models that quantified dead roots only within the species, not in composite samples with other species. The models were constructed for *Eriophorum vaginatum* and *Vaccinium myrtillus* using pure root samples from the site in Czechia in the *distant validation sample set VII*. The samples were collected at three different times of the growing season which allowed us to construct the calibration models on samples from one sampling time and validate them on samples from the same site but different sampling time (external validation). The models were then validated on living and dead roots of the given species from Finland (distant validation). For *Eriophorum vaginatum* the models were also validated on living roots from Canada, United Kingdom and Sweden (distant validation).

## Results

### Defining Root Types Based on FTIR Spectra

**FIGURE 2:**
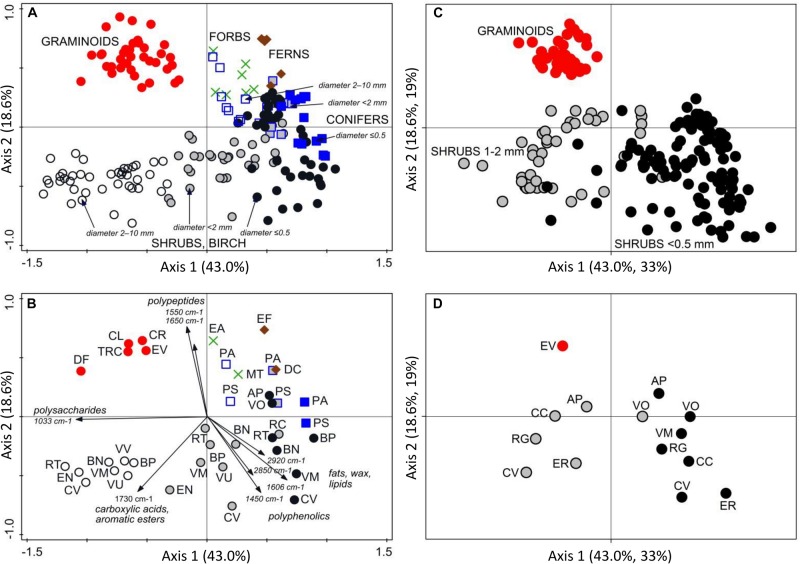
PCA analysis of FTIR spectra of plant roots. Ordination diagrams from principal component analysis (PCA) showing **(A)** variation in FTIR-derived chemistry of pure root samples from Finland (calibration and external validation datasets I-V; Table 1 and Figure 1), with **(B)** centroids for the different plant species and diameter class, and arrows showing the main FTIR absorption bands that defined the Axis 1 and 2. Pure roots from Canada, Czechia, Sweden and United Kingdom (distant validation sample set VII) were **(C)** projected into the ordination space defined by the samples from Finland, with **(D)** centroids for the different plant species and diameter class. Species abbreviations are in Table 1. Symbols for the root types: graminoids, red circles; forbs, green crosses; ferns, brown diamonds; conifers diameter ≤ 0.5 mm, blue squares; conifers diameter < 2 mm, gray squares; conifers diameter 2–10 mm, open squares; shrubs and birch diameter ≤ 0.5 mm, black circles; shrubs and birch diameter < 2 mm, gray circles; shrubs and birch diameter 2–10 mm, open circles.

**FIGURE 3:**
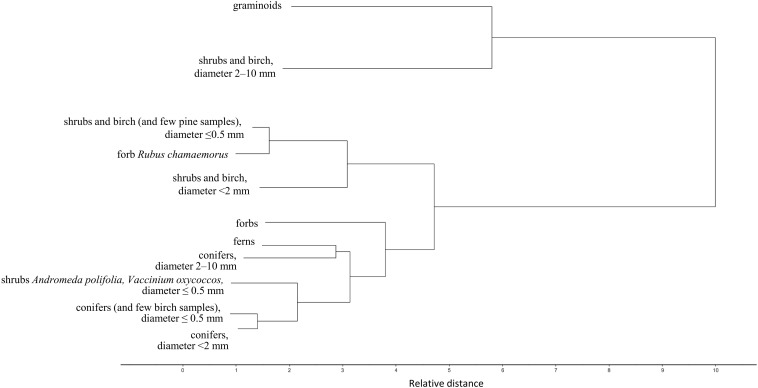
Cluster analysis of the plant root FTIR spectra. Dendrogram of the second derivative of the root FTIR spectra, calculated with Ward’s method and Squared Euclidean Distance, on pure root samples from Finland, the *calibration* and *external validation datasets I*-*V* (Table 1 and Figure 1). For simplicity only the main clusters are shown, not the individual samples.

**FIGURE 4:**
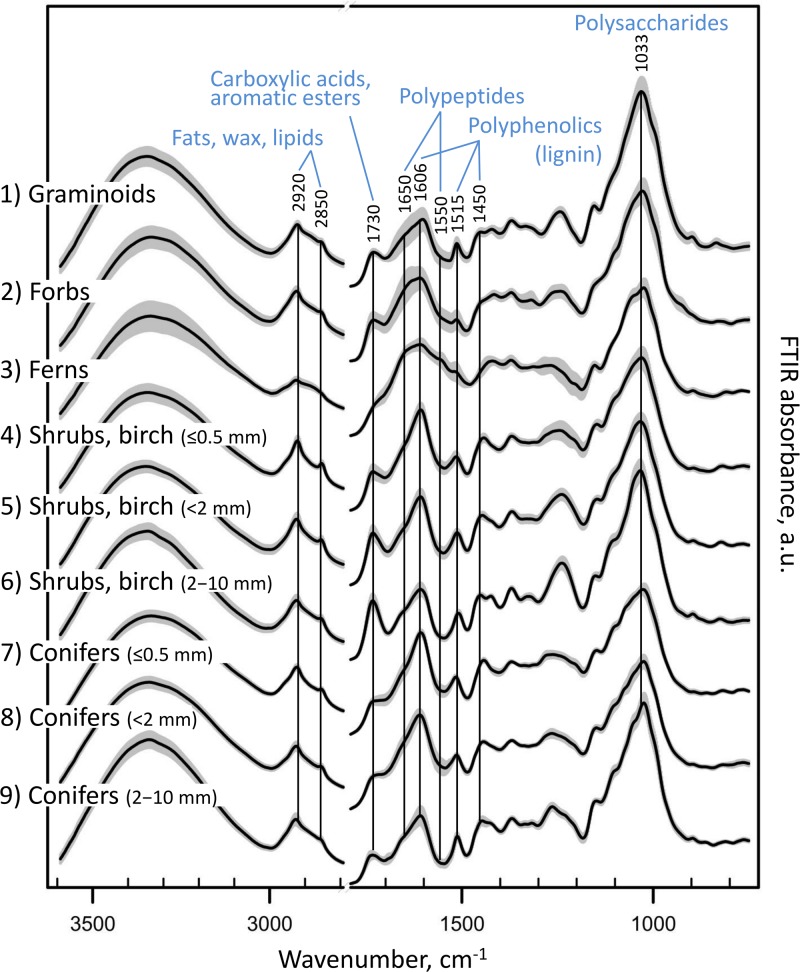
FTIR spectra of the different root types. FTIR absorbance spectra of pure root samples from Finland (calibration and external validation datasets I-V; Table 1 and Figure 1), grouped into nine root types: **(1)** graminoids; **(2)** forbs; **(3)** ferns; **(4)** shrubs and birch: very fine roots (diameter ≤ 0.5 mm); **(5)** shrubs and birch: fine roots (diameter < 2 mm); **(6)** shrubs and birch: coarser roots (diameter 2–10 mm); **(7)** conifers: very fine roots (diameter ≤ 0.5 mm); **(8)** conifers: fine roots (diameter < 2 mm); **(9)** conifers: coarser roots (diameter 2–10 mm). Black lines show means for each group, and gray area the standard deviation.

Plant roots were separated into nine root types, or root “chemotypes”, based on their FTIR spectra (Figures 2–4), roots of: (1) graminoids; (2) forbs; (3) ferns; (4) shrubs and birch: very fine roots (diameter ≤ 0.5 mm); (5) shrubs and birch: fine roots (diameter < 2 mm); (6) shrubs and birch: coarser roots (diameter 2–10 mm); (7) conifers: very fine roots (diameter ≤ 0.5 mm); (8) conifers: fine roots (diameter < 2 mm); (9) conifers: coarser roots (diameter 2–10 mm). Roots of graminoids with coarser roots of shrubs and birch differed from the other root types along the first PCA axis, which accounted for 43% of the variation in the root FTIR data (Figure 2). The first axis was largely defined by absorbance at 1033 cm^–1^, assigned to polysaccharides (relatively higher absorbance in graminoids and coarser shrub and birch roots). Woody (shrub and tree) roots differed from the herbaceous (graminoid, forb and fern) roots along the second PCA axis, which accounted for 19% of the variation in the root FTIR data. The second axis was defined by absorbance at 1650 cm^–1^ and 1550 cm^–1^ assigned to polypeptides (amide I and II; relatively higher absorbance in the herbaceous roots), and 1606 cm^–1^ and 1450 cm^–1^ assigned to polyphenolics (relatively higher absorbance in shrub and tree roots). The FTIR patterns related to root type were consistent across different sample sets (Figure 2).

Roots of the herbaceous plants showed rather high chemical variation: forbs and ferns were not similar to graminoids but rather grouped with the roots of conifers and fine or very fine roots of shrubs and birch (Figures 2, 3). Roots of forb *Rubus chamaemorus* were similar to roots of shrubs and birch and thus were added to shrubs and birch in the root type level calibration models. The other forbs and the ferns were more similar to conifers, but still formed distinct clusters (Figure 3).

**FIGURE 5:**
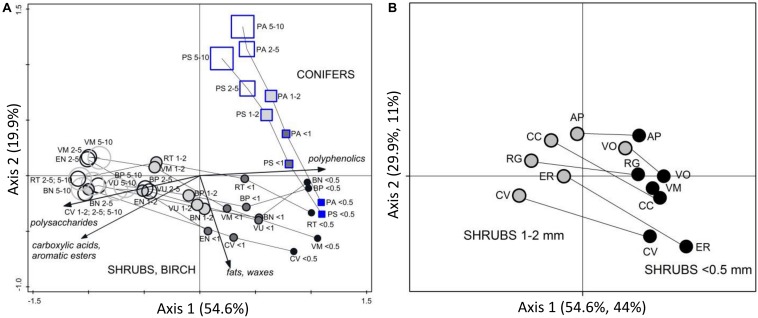
PCA analysis of FTIR spectra of woody roots of different diameter class. Ordination diagrams from principal component analysis (PCA) showing **(A)** variation in FTIR-derived chemistry of pure root samples of woody roots, in relation to root diameter. The roots were collected at one study site in Finland (drained bog Kalevansuo) in one date. Diameter classes: ≤ 0.5 mm, < 1 mm, 1–2 mm, 2–5 mm, 5–10 mm. Shrub and broadleaf tree species are shown by circles while conifer tree species are shown by squares. Pure shrub roots from Canada, Czechia, Sweden and United Kingdom (distant validation sample set VII) and their rhizomes (1–2 mm) were **(B)** projected into the ordination space defined by the samples from Finland, only the centroids for the different plant species and diameter class are shown. Species abbreviations are in Table 1.

Within woody species, our only broadleaf tree (birch *B. pubescens*) could not be distinguished from shrubs (Figure 2). Among shrubs, two species (*Andromeda polifolia* and *Vaccinium oxycoccos*) separated from others and grouped more closely with coniferous trees, forming however a distinct group in the cluster analysis (Figure 3). After testing different options we decided yet to add those to the other shrubs and birch in the root type level calibration models. Noteworthy, for all woody species, the within-species variation related to root diameter was in general higher than the difference from the other species (Figure 5). Conifers showed smaller within-species variation related to the root diameter than shrubs and birch (Figures 2, 3, 5). In general, the very fine roots (diameter ≤ 0.5 mm) of all the woody species were rather similar. With increasing root diameter the difference between shrubs and birch on one hand and conifers on the other hand increased. Fine and very fine roots were characterized by higher absorbance at 1606 cm^–1^, 1515 cm^–1^ and 1450 cm^–1^ cm^–1^ assigned to polyphenolics (lignin) as well as 2920 cm^–1^ and 2850 cm^–1^ assigned to aliphatic (wax, lipids) compounds. The coarser roots in turn were characterized by higher absorbance at 1033 cm^–1^ assigned to polysaccharides (Figures 2, 3, 5).

There were few trends in the variation in FTIR-derived root characteristics related to season, and these trends were marginal compared to the variation related to root type. The absorbance at wavenumbers assigned to polysaccharides tended to increase from early spring to summer and decrease in autumn, while absorbance assigned to polyphenolics had the opposite pattern (data not shown).

### Estimating Mass Proportions at the Level of Root Type

**FIGURE 6:**
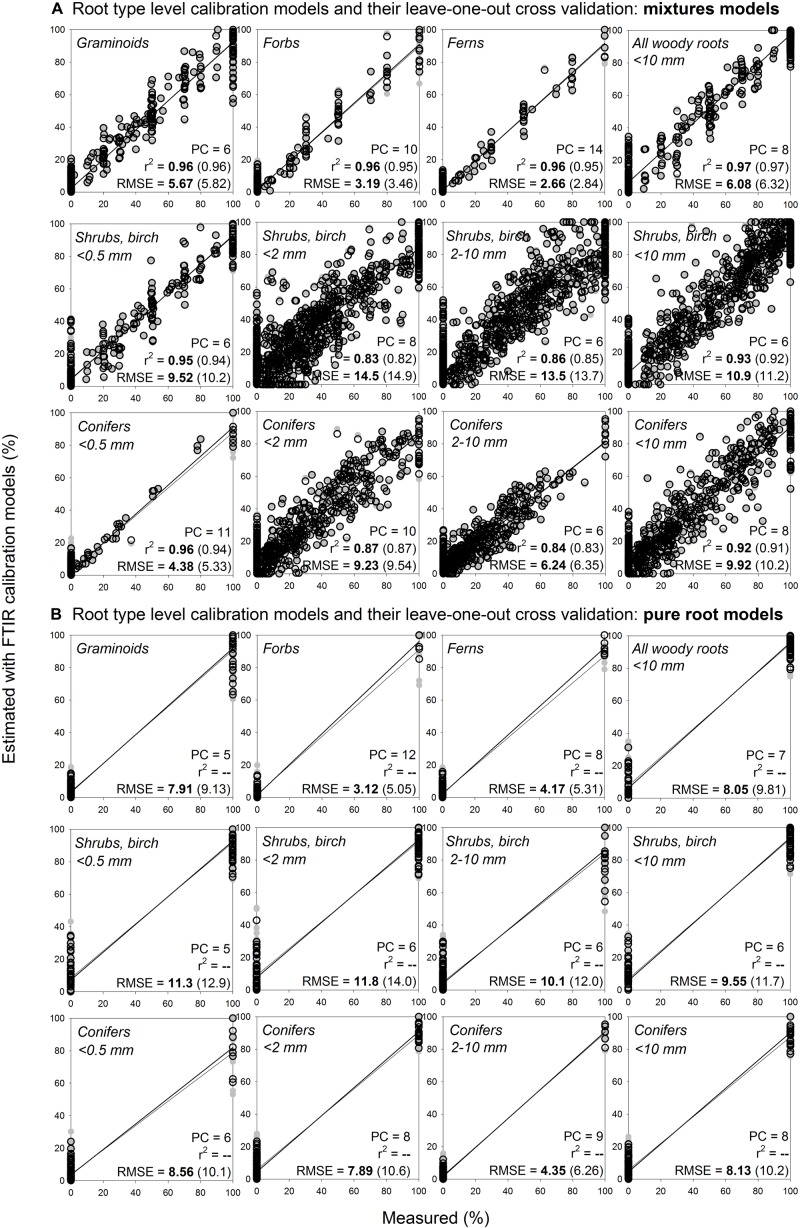
Root type level calibration models and their internal validation: comparison of mixtures models and pure roots models. Relationships between the measured percentage of roots of the specific root type in composite root samples and the percentage estimated with FTIR calibration models. **(A)** For calibration of the mixtures models for shrubs and birch diameter ≤ 0.5 mm and conifers diameter ≤ 0.5 mm, the calibration and external validation sample sets I, III, V and VI (Table 1 and Figure 1) were used, *n* = 333. For calibration of the mixtures models for all the other root types, samples from the calibration and external validation sample sets I–VI were used, *n* = 1218. **(B)** For calibration of the pure root models, only the pure roots (no mixtures) of the sample sets specified for **(A)** were used, *n* = 128 for woody roots diameter ≤ 0.5 while *n* = 216 for all the other root types. PC is the number of factors (“principal components”) included in the calibration models and RMSE is the root mean square error. Calibration values are shown by black open symbols with RMSE and *r*^2^ in bold letters, the internal full-cross validation values are shown by gray symbols with values of RMSE and *r*^2^ in parentheses.

Using root mixtures, calibration models for graminoids, forbs, ferns, and all woody roots (diameter < 10 mm) had RMSE of 5.7, 3.2, 2.7, and 6.1%, respectively, and *r*^2^ 0.96–0.97 (Figure 6A). Calibration models sorting woody roots to conifers and shrubs with birch also performed reasonably well, with RMSE 9.9–10.9% and *r*^2^ 0.92–0.93. Calibration models further sorting the woody roots based on their diameter (very fine ≤ 0.5 mm, fine < 2 mm, coarser 2–10 mm) had RMSE of 4.4–9.2% for conifers and 9.5–14.5% for shrubs with birch, with *r*^2^ 0.83–0.96. Calibration models that included only pure root samples had RMSEs comparable to the mixtures models, often with even lower number of factors (PCs) used (Figure 6B). For all the models, the internal validation outcomes were in similar range as for the calibrations (Figures 6A,B).

**FIGURE 7:**
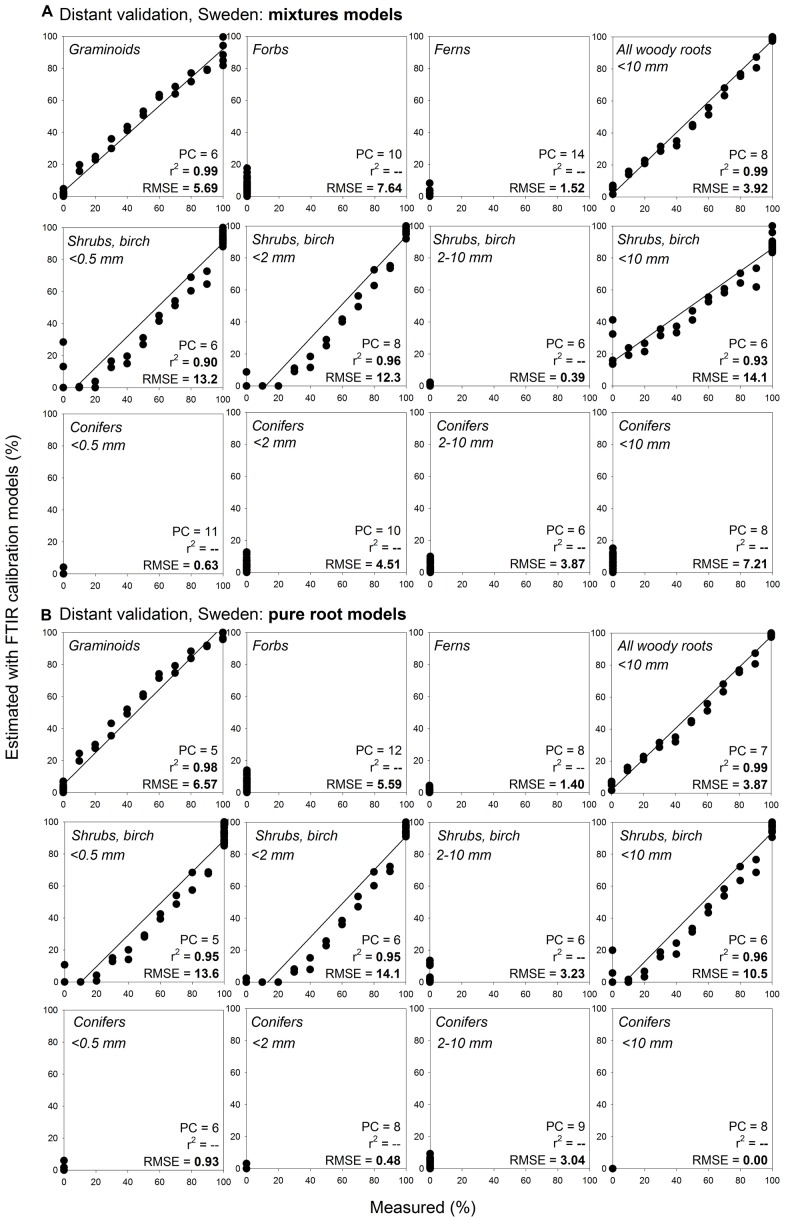
Distant validation, Sweden, of the root type level calibration models: comparison of estimates using mixtures models and pure roots models. Relationships between the measured percentage of roots of the specific root type in composite root samples and the percentage estimated using FTIR calibration models: comparison of estimates using **(A)** mixtures models and **(B)** pure roots models. The calibration models are presented in Figure 5. Distant validation of the models show samples from wet fen site in Sweden (distant validation sample set VII, Table 1) that included plant species present in the calibration, *n* = 44. PC is the number of factors (“principal components”) included in the calibration models and RMSE is the root mean square error. Distant validation of the pure root models on the remaining samples from the distant validation sample set VII is shown in Supplementary Figures S1–S3, S13.

Distant validation of the root type level models provided acceptable predictions even at far distance sites, with predictions being, similarly to the calibration models, generally somewhat better for herbaceous plant roots than for the woody plant roots (Figure 7, Supplementary Figures S1–S3). All woody roots present in the distant validation dataset were of diameter ≤ 0.5 mm and woody models for this diameter class generally provided more precise predictions than the woody models that contained all diameter classes < 10 mm. Noteworthy, root type level calibration models that included only pure root samples yielded comparable or better predictions than the models constructed using the mixtures. This finding was consistent across the different distant validation sites (Figure 7, Supplementary Figures S1–S3).

Using pure root models, the root type level predictions for sites with species that were included in the calibration models had RMSE of 0.0–14.1% at the Swedish site (Figure 7B), or 3.3–20.1% at the Czech site (Supplementary Figure S1B; if for woody roots only the models for very fine diameter were considered then RMSE ranged 3.3–7.9%). Predictions for roots from the sites in Canada and United Kingdom that also included shrub species not present in the calibration models had RMSE of 0.0–17.1% (Supplementary Figures S2B, S3B; if for woody roots only the models for very fine diameter were considered then RMSE ranged 1.4–15.8%).

### Estimating Mass Proportions at the Level of Plant Species

#### Herbaceous Species: Graminoids, Forbs and Ferns

Calibration models for roots of 8 herbaceous species had RMSE ranging from 2.6% to 6.2% and *r*^2^ from 0.97 to 0.99, with internal validation outcomes in similar range (Supplementary Figure S4). However, the calibration models did not yield reliable estimates of herbaceous species composition during the external and distant validation (results not shown). For graminoids, the RMSEs ranged from 0–10% when models correctly estimated that the graminoid species roots were not forb or fern species, to 30–70% when the models did not distinguish the graminoid species *Carex lasiocarpa*, *Carex rostrata* or *Eriophorum vaginatum* from each other. For forbs, the only species present in the external validation dataset was *Menyanthes trifoliata* and its roots were successfully recognized by the calibration model for the species (RMSE 11.0%). There were two species present in the external validation dataset but not included in the calibration models, and those species were not estimated as “non-present” (prediction 0%) but rather partly overlapped with similar species: *Trichophorum cespitosum* was estimated as being 38% *Carex lasiocarpa* or 44% *Eriophorum vaginatum*, while *Equisetum fluviatile* was estimated as 62% *Dryopteris carthusiana.*

#### Woody Species: Shrubs, Broadleaf Tree, Coniferous Trees

Narrow calibration models for shrubs and trees at the level of plant species and diameter class (diameter < 2 mm and 2–10 mm) fitted the calibration samples well with RMSE 2.4–6.5% and *r*^2^ 0.92–0.97, and internal validation outcomes in similar range (Supplementary Figure S5). The calibration models well distinguished and quantified even species of the same genus: *Betula nana* and *Betula pubescens*, *Vaccinium myrtillus* and *Vaccinium uliginosum*.

However, the narrow calibration models did not yield reliable estimates during the external validation on roots of same species and diameter, with RMSE 7.2–27.2% for species level estimates and 8.1–41.3% for diameter class level estimates, and *r*^2^ 0.02–0.85 (Supplementary Figure S6). When the models were applied on two species that were not included in the narrow calibration models, *Picea abies* and *Vaccinium vitis-idaea*, those species were not estimated as “non-present” but largely overlapped with the other woody species (data not shown).

**FIGURE 8:**
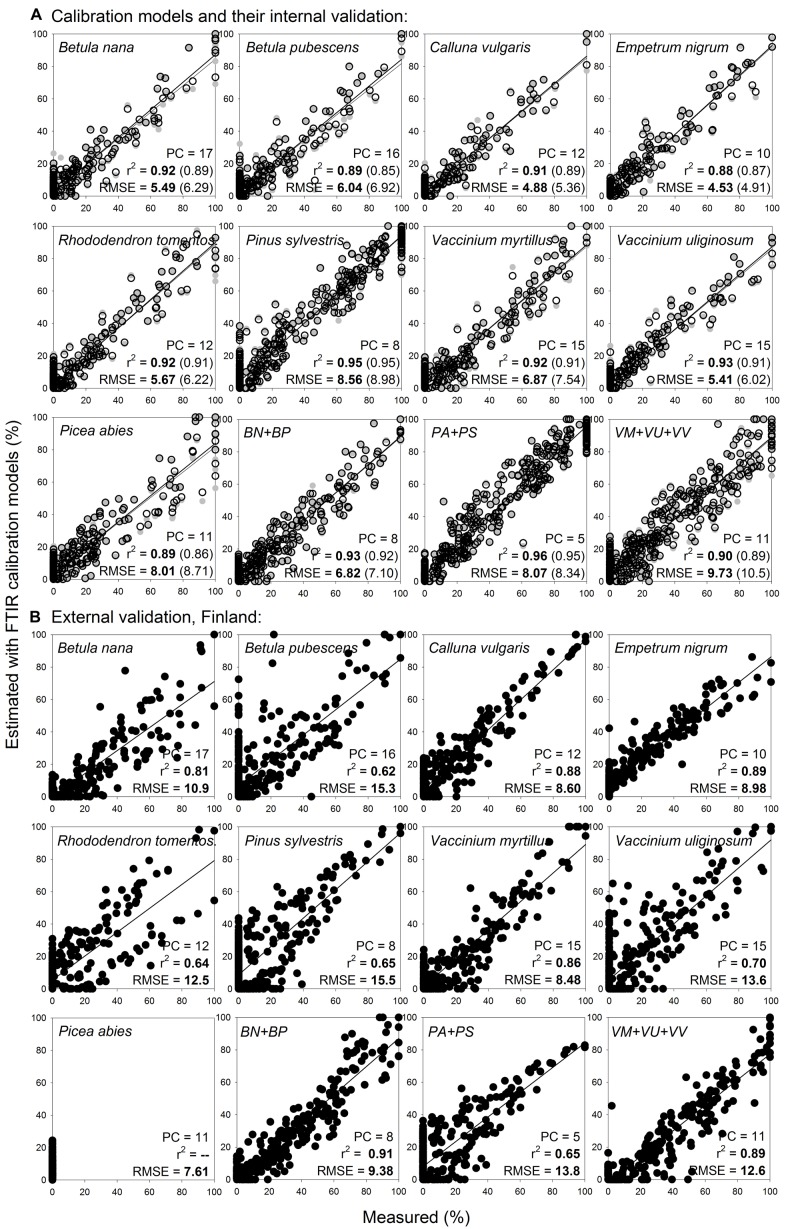
Species level calibration models (broader) for woody roots, with external validation on newly collected samples. Relationships between the measured percentage of roots of the different woody plant species in composite root samples and the percentage estimated with FTIR calibration models. **(A)** For calibration, samples from calibration sample set II (Table 1 and Figure 1) that included fine (< 2 mm) and coarser (2–10 mm) roots were used, *n* = 518. **(B)** External validation of the models show samples from external validation sample set IV, that included woody roots of the same species and diameter classes as present in the calibrations, *n* = 367. PC is the number of factors (“principal components”) included in the calibration models and RMSE is the root mean square error. Calibration values are shown by black open symbols with RMSE and *r*^2^ in bold letters, the internal full-cross validation values are shown by gray symbols with values of RMSE and *r*^2^ in parentheses, and the external validation values are shown by black full symbols. Species abbreviations are in Table 1. External validation of the models on very fine (≤ 0.5 mm) woody roots of the same species is shown in Supplementary Figure S7.

Broader calibration models for woody roots at the level of plant species, that compared to the narrow models covered broader variation for each species, fitted the calibration samples with RMSE 4.5–8.6% and *r*^2^ 0.88–0.95, and internal validation outcomes in similar range (Figure 8A).

External validation of the broader models on roots of the same species and diameter provided better estimates than did the narrow models, with RMSE 8.5–15.5% and *r*^2^ 0.64–0.89 (Figure 8B). For several species, most obviously for *Rhododendron tomentosum*, the estimates showed a trend of forming two clusters with different slopes in their regression lines. The clusters were related to the two diameter classes (diameter < 2 mm and 2–10 mm) with underestimated proportions of the fine diameter (< 2 mm) class (Figure 8B). Calibration models that merged roots of the same genus, *Betula nana* with *Betula pubescens, Vaccinium myrtillus* with *Vaccinium uliginosum* and *Vaccinium vitis-idaea*, or the two coniferous tree species *Pinus sylvestris* with *Picea abies*, provided marginal improvement of the external validation predictions compared to the species-level models (Figure 8).

External validation of the broader calibration models on very fine woody roots (diameter < 0.5 mm), provided reliable estimates only for *Calluna vulgaris* with RMSE 5.5%, for the other woody species RMSE was 12.5–47.5% (Supplementary Figure S7). Distant validation of the broader calibration models on very fine roots again provided acceptable estimates only for *Calluna vulgaris* with RMSE 14.3% (not shown).

Noteworthy, calibration models focusing on very fine woody roots that were constructed including only a limited number of pure root samples (no mixtures) fitted the root samples well with RMSE 0.9–6.2% for the species or grouped species, with internal validation outcomes in similar range (Supplementary Figure S8A). We had only limited number of samples of the given species and diameter class available for external and distant validation of these models. Still, the validation results indicate that these models provide better estimates for the very fine woody roots than the broader models above that included roots of different diameter classes < 10 mm. Distant validation of the models provided estimates with RMSE 4.3–23.2% (Supplementary Figures S8B,C).

#### Herbaceous and Woody Species Together in Calibration Models

Merging herbaceous and woody roots in general calibration models (Supplementary Figure S9) did not decrease the prediction ability of the models. Compared to the models constructed separately for herbaceous and woody species (Figure 8 and Supplementary Figure S4), the calibration and internal validation RMSE of the general models were even lower as the two types of roots (woody vs. herbaceous) were well distinguished. External and distant validation of the general models provided similar estimates as did the herbaceous root models for herbaceous roots and the woody root models for woody roots (not shown). Similarly to the woody models, however, the general models calibrated on woody samples with different diameter roots (diameter < 10 mm) did not provide reliable estimates for the very fine woody roots (diameter ≤ 0.5 mm).

**FIGURE 9:**
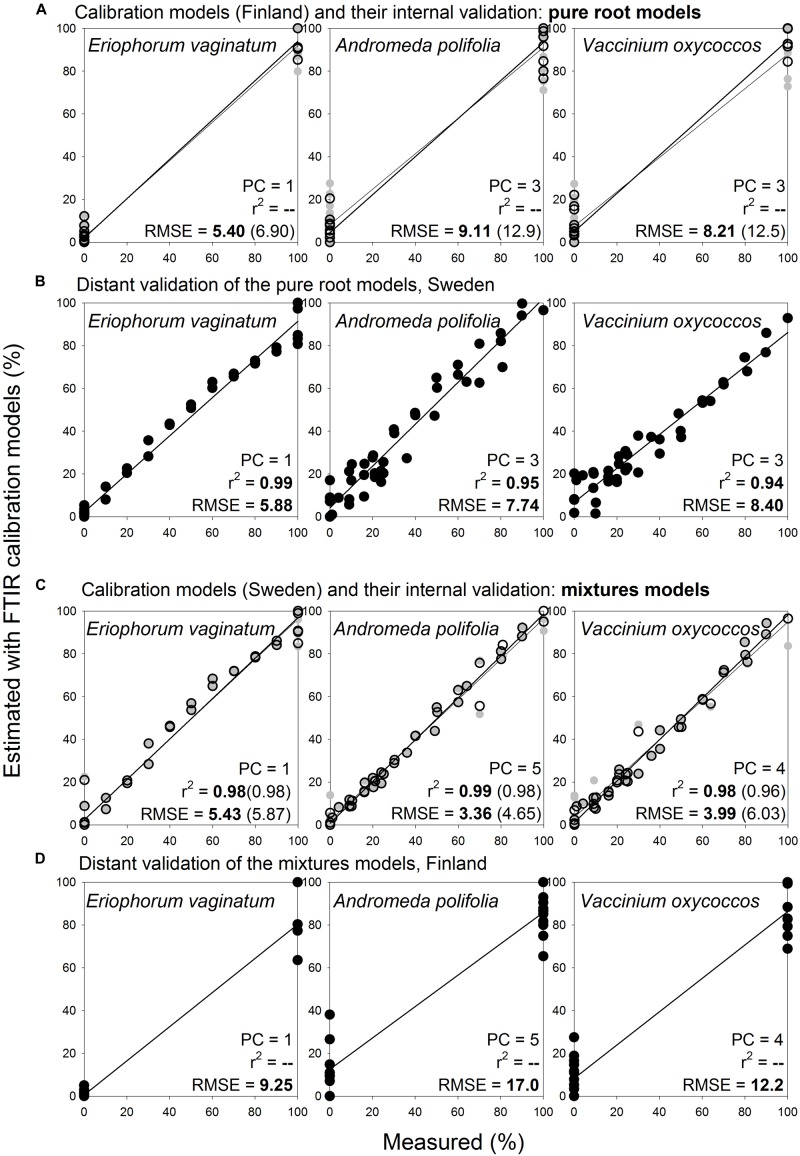
Species level calibration models, comparison of results obtained with pure root and mixtures models for three species, distant validation between Finnish and Swedish sites. Relationships between the measured percentage of roots of the different plant species in composite root samples and the percentage estimated with FTIR calibration models. **(A)** For calibration, pure roots of the selected herbaceous and woody roots of the calibration and external validation sample sets I and V (Table 1) were used, *n* = 25. **(B)** Distant validation of the models show samples (both pure roots and their mixtures) of the same species as used in the calibrations, from the site in Sweden in the distant validation sample set VII, *n* = 44. **(C)** Calibration models were constructed using the same samples described for **(B)**. **(D)** Distant validation of the models was done using the same samples described for **(A)**. PC is the number of factors (“principal components”) included in the calibration models and RMSE is the root mean square error. Calibration values are shown by black open symbols with RMSE and *r*^2^ in bold letters, the internal full-cross validation values are shown by gray symbols with values of RMSE and *r*^2^ in parentheses, and the distant validation values are shown by black full symbols.

**FIGURE 10:**
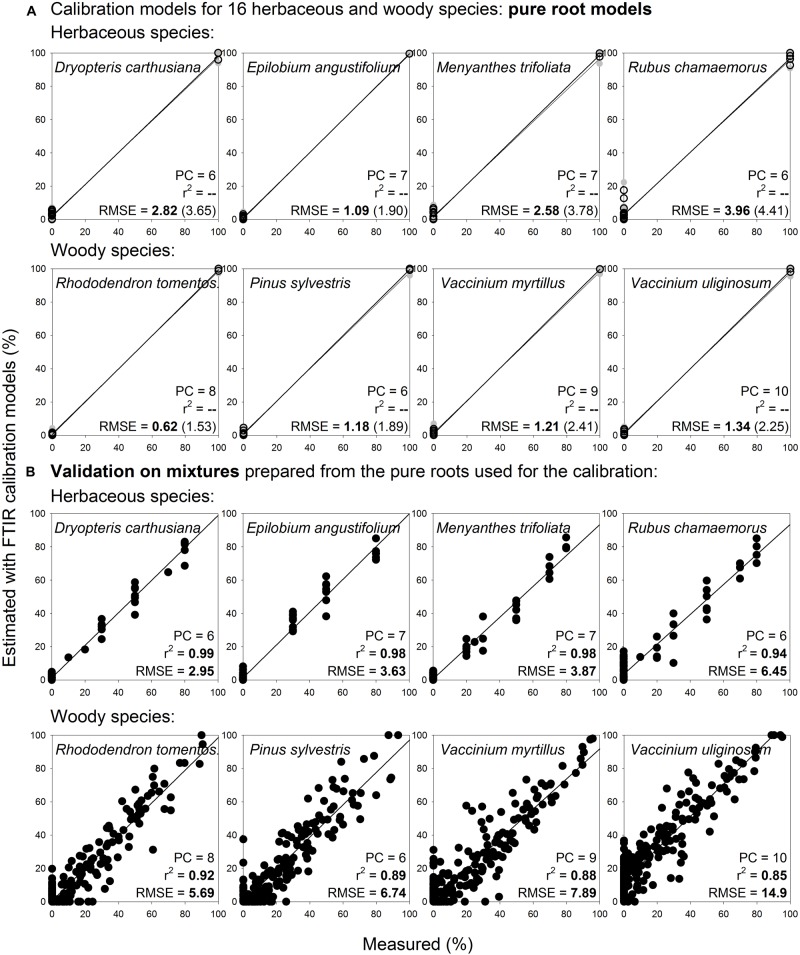
Species level calibration models constructed on pure roots of 16 species and their validation on multispecies mixtures prepared from the pure roots used for the calibration. Relationships between the measured percentage of roots of the different plant species in composite root samples and the percentage estimated with FTIR calibration models. **(A)** For calibration, pure roots of 16 herbaceous and woody species of the calibration and external validation sample sets I and IV (Table 1) were used, only living roots were included, *n* = 38. **(B)** Validation of the models show artificial mixtures containing known mass proportions of roots of the given species, that were prepared from the pure roots used for the calibration, *n* = 466. PC is the number of factors (“principal components”) included in the calibration models and RMSE is the root mean square error. Calibration values are shown by black open symbols with RMSE and *r*^2^ in bold letters, the internal full-cross validation values are shown by gray symbols with values of RMSE and *r*^2^ in parentheses, and the distant validation values are shown by black full symbols. Only graphs for 8 species are shown, graphs for the remaining 8 species are shown in Supplementary Figure S10.

Noteworthy, pure root calibration models that were constructed for selected herbaceous and woody roots (3 species: *Eriophorum vaginatum*, *Andromeda polifolia*, *Vaccinium oxycoccos*; Figure 9A) provided very good estimates for the same species and diameter class during distant validation, with RMSE 5.9–8.4% (Figure 9B). Prediction abilities of these three-species “pure root” models were comparable with the predictions using “mixtures” models (Figures 9C,D). Furthermore, when we applied this approach on 16 species of herbaceous and woody plants, the pure root models (Figure 10A) provided very good estimates of proportions of the given species in composite samples (prepared from the pure roots used for the calibration), with RMSE 3.0–14.9% (Figure 10B, Supplementary Figure S10).

### Dead Roots

Compared to the living roots of the given species, the field-dead roots showed a pattern of relatively higher absorbance at FTIR regions assigned to polyphenolics (1606 cm^–1^, 1515 cm^–1^, 1450 cm^–1^) and polypeptides (1650 cm^–1^ and 1550 cm^–1^). In contrast, lower absorbance at FTIR regions assigned to polysaccharides (1033 cm^–1^), aliphatics: fats, wax, lipids (2920 cm^–1^ and 2850 cm^–1^) and carboxylic acids or aromatic esters (1730 cm^–1^) was found for field-dead roots.

There was only negligible change in FTIR-derived chemistry from living to artificially dead roots of *Vaccinium myrtillus* and *Pinus sylvestris* from the greenhouse experiment. Still, similarly to the field-dead roots, there was a trend of relatively higher absorbance at FTIR regions assigned to polyphenolics and lower absorbance at FTIR regions assigned to polysaccharides in the artificially dead roots (Supplementary Figure S11).

Within species, the calibration models constructed on pure root samples provided very good estimates of field-dead roots of *Vaccinium myrtillus*, with external validation RMSE 2.7% and 8.4% (Supplementary Figure S12). The artificially dead roots did not represent well the field-dead roots and were estimated as 100% living during the distant validation (RMSE 100%), while the living roots were estimated correctly, with RMSE 4.5% (not shown). For *Eriophorum vaginatum*, the calibration models estimated field-dead roots correctly with external validation RMSE 6.7% and 13.2% (Supplementary Figure S12). Distant validation provided correct estimates of field-dead and/or living *Eriophorum vaginatum* roots for four of seven tested study sites, with RMSE < 10%, while for the three remaining sites the estimates were unacceptable with RMSE 30–40% (not shown).

The root type level models showed somewhat different predictions for living (Supplementary Figure S1) and field-dead (Supplementary Figure S12) roots of *Vaccinium myrtillus* during distant validation at the Czech site, the field-dead roots were estimated like conifers rather than shrubs.

## Discussion

### Quantification at the Level of Plant Functional Type or Root Type Was Possible Even for Distant Samples; Simple Identification of Graminoids

We were able to construct generally applicable FTIR calibration models for quantification of roots of the main PFTs of northern peatlands: graminoids, forbs, ferns, shrubs (including a broadleaf tree), and coniferous trees. It was also possible to distinguish diameter classes for roots of woody plants. The models estimated mass proportions of these root types in composite samples with relatively low error, even for roots from far distance sites that were of different plant species than those included in the calibration models. These results provide robust support to the hypotheses that roots of peatland PFTs (H1) and diameter classes (H2) can be distinguished and quantified using FTIR spectra. Such root-type level models have to our knowledge not been built before, and could be more widely applicable than species-level models.

Graminoid roots clearly differed from all the other root types. This enabled their reliable quantification in composite samples using the calibration models. They could also be identified using PCA or cluster analysis, in our extensive dataset with 100% success. Forbs and ferns belong to herbaceous plants, but their root FTIR-derived chemistry was quite different from graminoids. Accordingly, Legner et al. (2018) showed clear separation of four graminoid species from a dicot species using cluster analysis of their root FTIR spectra. Forbs and ferns showed similarity with the roots of coniferous trees and fine or very fine roots of shrubs and birch. Still they formed separate clusters, which is a precondition for correct quantification by the calibration models. Yet, the distant validation for non-presence of forbs and ferns in root samples indicated that there may be some overlap with trees and shrubs in the estimates. In reality, the accuracy of the estimates can be improved with information on plant species or PFT presence at target sites, which is easily based on aboveground observations. Thus, if there is no evidence of, e.g., fern presence, fern models need not be applied at all.

In earlier studies all herbaceous roots, and sometimes all ground vegetation roots have usually been pooled, irrespective of whether NIR spectra (Lei and Bauhus, 2010; Domisch et al., 2015; Finér et al., 2017) or visual identification (Bhuiyan et al., 2017) was applied. Our plant functional type or root type level calibration models offer a possibility for more detailed identification of understory vegetation roots. This is important since it will make it possible to produce data for ecosystem models to represent PFTs correctly also belowground. This will allow, for instance, accounting for the different turnover rates (Gill and Jackson, 2000) and decomposability (Straková et al., 2010, 2012) of the PFTs. In peatlands, the high water-table levels and soil anoxia largely shape the root depth distributions of plant species and PFTs (Murphy et al., 2009a, b; Murphy and Moore, 2010). Thus, these distributions may greatly differ from patterns typical in oxic mineral soils (e.g., Westoby and Wright, 2006). Consequently, estimation of the rooting patterns of PFTs cannot be based on insights from mineral soils.

### Diameter Class Affected Estimates of Conifers, Shrubs and Birch, but Not Woody Roots in Total

The diameter-related results on woody roots partly disagree with our initial hypothesis stating that roots of different diameter class can be distinguished and quantified using FTIR spectra (H2). Fine and coarser roots showed different FTIR signatures, as we expected. However, the diameter class differences overrode the between-species chemical variation and negatively affected estimates at the level of species or PFT (coniferous tree vs. shrubs with birch). Still, it was well possible to quantify woody roots in total. Our results demonstrated clear diameter class differences in woody roots, and ability of the models to quantify the different classes in artificial mixtures. However, we must conclude that in practise, we are not able to quantify their proportions in field samples, where there are no clearly defined classes (only) present, but a continuum of roots of varying diameters. The practical conclusion is thus that when applying the models for identifying roots in field samples we should focus on very fine or fine roots only. Roots coarser than 2 mm should be manually removed, if present, and analyzed separately. The good thing is that coarse roots are rather easily separated, unlike fine roots. The unfortunate thing is that the compositional differences between the roots of conifers and shrubs with birch decrease with decreasing root diameter and consequently, also the ability of calibration models to correctly distinguish and quantify these root types decreases.

The diameter-related chemical variation that we present for roots is supported by earlier findings that show increase in polyphenolic (lignin) and nutrient concentration and decrease in polysaccharide (cellulose) concentration with decreased root diameter (Pregitzer et al., 2002; Thomas et al., 2014; Zhang et al., 2014). They are also in line with the woody plant chemistry aboveground: similar differences were observed for branch litter, with finer branches having higher concentration of lignin and nutrients and lower holocellulose concentration compared to coarser branches (Vávřová et al., 2009; Straková et al., 2010).

Compared to branches or coarser roots, the very fine root chemistry and consequently, FTIR signature is, however, additionally affected by mycorrhizal colonization (Pena et al., 2014), which may also complicate the root species identification. While there is little information from peatlands, in general both birch and pine species have shown 100% mycorrhizal colonization rate and no secondary xylem or continuous cork layer for the finest (branching order 1) roots (Guo et al., 2008), which may make the root FTIR signatures rather similar between species. In the same study birch then showed decrease in mycorrhizal colonization and increase in secondary xylem and continuous cork layer from branching order 2 and pine from branching order 3, providing support for our finding that species-specific compositional patterns become more evident with increasing root thickness. Other studies also reported some overlap of NIR-derived chemistry of coniferous and broadleaf tree fine roots that was reflected in prediction abilities of the calibration models (Lei and Bauhus, 2010; Domisch et al., 2015; Finér et al., 2017).

Concerning herbaceous plants, we did not analyse diameter effect as the actual diameters were not determined for all samples. The roots were all of diameter < 2 mm, thus belonging in the fine root class. This class, however, included also very fine roots, if they were formed by the given species. In another study, species-dependent diameter class differences were observed for graminoid and forb forage species (White et al., 2011). Two of their four species had largely the same composition regardless of diameter, while the other two showed different composition for roots of diameter < 1mm and coarser than 1mm. Finer roots showed higher absorbance at wavenumbers assigned to polyphenolics relative to polysaccharides, in line with our results for woody roots, but higher absorbance at wavenumbers assigned to carbonyls, which is in contrast with our results for woody roots that showed higher absorbance at this region in the coarser diameter class only. The finest part of the roots with yet undifferentiated cells, the root tips, had to be removed in Meinen and Rauber (2015) and Legner et al. (2018) to get full species differentiation of various segments of forage roots by cluster analysis of their FTIR spectra. Thus, we may conclude that diameter variation possibly affected the model fit to graminoids.

### Quantifications at Species Level Are Not Routinely Applicable in Field Conditions

The results on species level quantification do not fully support our hypothesis that roots of all peatland plant species can be distinguished and quantified using FTIR (H1). We were not able to clearly distinguish and quantify species belonging to the same PFT. The within-species (intraspecific) heterogeneity captured by FTIR was higher for the species sampled at different sites and/or sampling times than the between-species (interspecific) chemical variation, limiting routine application of the species-level models in field conditions.

This outcome seems to be in contrast with a study that examined FTIR spectra of five herbaceous agriculture plants and concluded that the roots of the same species are similar despite differences in climate, soil and fertilization, while important differences were noted between roots of different species (White et al., 2011). Several other FTIR or NIR studies that quantified roots of closely related species in mixtures also ended in more optimistic conclusions concerning the predictive power than we (Rumbaugh et al., 1988; Roumet et al., 2006; Picon-Cochard et al., 2009; Naumann et al., 2010; Kusumo et al., 2011; White et al., 2011; Meinen and Rauber, 2015; Legner et al., 2018; Streit et al., 2019; Lei and Bauhus, 2010; Domisch et al., 2015; Tong et al., 2016; Finér et al., 2017; Picon-Cochard et al., 2009). However, to our knowledge, none of the earlier studies tested their models on newly collected independent samples. Also our models and their internal cross-validation results look very promising as such. However, when applied on newly collected samples of the same species coming from various sites, the models did not reliably distinguish and quantify the species. We thus have to conclude that despite of our effort to include high natural variation in our samples, the calibration models cannot be successfully applied on new samples outside the calibration dataset. We suggest that the previous studies using the same methodology were overoptimistic, and the models should not be used for routine application without careful external validation. They may, naturally, still be valid for the specific study settings that they were created for.

### Dead Roots Differ From Living, but the Calibration Models Are Not Routinely Applicable in Field Conditions

We found FTIR-derived differences between living and dead roots and demonstrated the potential to quantify the dead and living roots within the given species. These results provide robust support to the hypotheses stating that proportions of dead and living roots can be quantified using FTIR, assuming that dead and living roots have different FTIR signatures (H3). However, models estimating dead root proportions only within a given species are insufficient for routine application in field conditions when multiple species are present. Theoretically, if combined with pure roots of other species, the models could be reconstructed to quantify proportions of dead roots in multiple species composite samples, and this will be the direction of our further work.

The need to include also dead roots in the calibration models in field studies has been concluded earlier (Lei and Bauhus, 2010). Still, so far only Picon-Cochard et al. (2009) attempted to quantify the proportions of dead and living roots in composite samples. They constructed models for separation of artificially produced, 1 and 2 months old dead roots, in mixtures of 5 graminoid species, with RMSE of one-leave out full cross validation 15%. Their mixtures were, however, prepared from a bulk sample for each species and living or dead root variant and thus are unlikely to give reliable estimates outside their experimental conditions.

Dead roots in field conditions may vary greatly in time passed since the root death, from recently dead to dead for years, in anoxic peat soils actually even millennia. This, in combination with the root initial chemistry and the environmental characteristics, determines their decomposition stage and thus their FTIR signatures (e.g., Duboc et al., 2012). Unfortunately, our experimental production did not yield suitable samples for the calibrations, the artificially dead roots did not represent well the field-dead roots. This means that one of the major challenges in creating dead root models is being able to harvest representative materials. Yet, with the pure root approach this is made at least a bit easier.

### Pure Root Models Are More Practical Than the Traditional Models Constructed on Root Mixtures

Our results confirm the extreme power of FTIR with chemometrics to distinguish and quantify different substrates mixed into a composite sample. We present distinction and quantification of even 16 substrates. This is, as such, nothing new. Noteworthy, we demonstrate that the substrates can be successfully quantified in composite samples using calibration models that are constructed on pure substrates only, without the need to prepare artificial mixtures of the different substrates for the model calibration. This finding has several practical implications for studies dealing with fine or very fine plant roots:

1) Significantly reduced amount of root material that needs to be collected for model calibration.
2) Consequently, possibility to cover higher within-species (intraspecific) heterogeneity, without the need to increase sample amount by pooling roots for each species.
3) Flexibility to add/remove particular sample type (e.g., species, or diameter class) from calibrations.
4) Reducing technical errors introduced by weighing and mixing the samples for mixtures

For example, 45 g of dry roots were used for creating narrow 5-species NIR mixtures models by Tong et al. (2016). A minimum of 500 mg dry root material was necessary to record the NIR spectrum of one sample (Tong et al., 2016; Lei and Bauhus, 2010). Such amount is quite hard to harvest in the very fine root diameter. Using FTIR-ATR method instead of NIR already significantly reduces the amount of roots needed to about 5–10 mg of dry root material per sample. Combined with our pure root approach, 5-species model covering sufficient within-species variation (10 samples per species) could be achieved with only 250–500 mg dry root material (5 species × 10 samples per species × 5–10 mg per sample).

Natural variation makes the difference between our attempts to quantify roots grown in field conditions, and roots produced in controlled greenhouse experiment, or even more clearly, between strictly defined chemicals. We need to capture the natural variation into the calibration sample library to make reliable predictions for new samples. Natural variation may be captured by well designed sampling, following objectives of the particular study, and sufficient number of samples per each species. However, most of the published root studies (including ours, to a large extent) needed to pool the collected roots to obtain enough material for creating the artificial mixtures. The only study that clearly stated that their mixtures were prepared from different individuals, not pooled samples, is the study by Lei and Bauhus (2010). The pure root approach allows us to cover much more within-species variation for more robust calibration models.

Previous studies recommended to increase the prediction quality of the calibration models by extending the calibration sample size by creating more artificial mixtures (e.g., Lei and Bauhus, 2010; Meinen and Rauber, 2015), giving the impression that the more mixtures, the better.

We had two assumptions based on earlier work that we took for granted when beginning our research:

– For successful quantification of species in composite samples, we need to achieve all possible combinations of the species in artificial mixtures for calibration,
– The more mixtures the better.

Now we argue that both of these were in fact wrong. We do agree that increasing the calibration sample size improves the models. However, we argue that while mixtures improve the fit of the calibration models, they do not improve the essential, which is the prediction ability of the models. Mixtures are basically just re-runs of samples that were used for the mixtures preparation. Instead of creating the artificial mixtures with all possible combinations of the species, we now suggest a different way of building models for routine applications in field conditions. That is creating an extensive root spectra database covering the species (that may be used to represent PFTs) and root types with several independent samples, and then selecting suitable samples from the database for each specific research goal using the pure root approach. This approach leaves the flexibility for selecting the combination of species or sample types best suited for each application, instead of being stuck with mixtures that include also irrelevant material that may distort the analyses. Seasonal variation in root chemistry (Lei and Bauhus, 2010; Kaštovská et al., 2018) should also be considered; either by including it extensively in the calibration sample library, or sampling at the same season as the forthcoming samples to be estimated by the models are to be collected.

## Conclusion

Our results confirm the extreme power of FTIR with chemometrics to distinguish and quantify different substrates mixed into composite samples. Noteworthy, we demonstrate that the substrates can be successfully quantified in composite samples using calibration models that are constructed on pure substrates only, without the need to prepare artificial mixtures with varying concentrations of the different substrates for calibrations of the models.

We were able to construct generally applicable FTIR calibration models for quantification of roots of the main PFTs of northern peatlands: graminoids, forbs, ferns, shrubs (including a broadleaf tree), and coniferous trees.

More detailed root quantifications, e.g., at species level or distinguishing dead roots from living, are in field conditions restricted by natural variation in root chemistry, and unclear boundaries between the different root classes. We were not yet able to construct such models for general application in field conditions, although we present a possibility for further development of such models using the pure root approach.

## Data Availability Statement

The datasets generated for this study are available on request to the corresponding author.

## Author Contributions

RL brought the initial idea for this study. PS, TL, KE, KM, and RL planned and designed the research, at various stages of this study. PS, TL, JA, NI, PL, KE, and RL participated on root sample collection and processing and/or FTIR measurements. NI and PL analyzed subsets of the data in their M.Sc. theses. PS completed and organized the final dataset, performed all the data analyses and prepared all the figures and the tables for this manuscript and wrote the manuscript, with inputs from RL and helpful comments from the other co-authors.

## Conflict of Interest

The authors declare that the research was conducted in the absence of any commercial or financial relationships that could be construed as a potential conflict of interest.

## References

[B1] Bellon-Maurel V. McBratney A. (2011). Near-infrared (NIR) and mid-infrared (MIR) spectroscopic techniques for assessing the amount of carbon stock in soils–Critical review and research perspectives. *Soil Biol. Biochem.* 43 1398–1410.

[B2] Bernard J. M. Solander D. Kvet J. (1988). Production and nutrient dynamics in Carex wetlands. *Aq. Bot.* 30 125–147.

[B3] Bhuiyan R. Minkkinen K. Helmisaari H. Ojanen P. Penttilä T. Laiho R. (2017). Estimating fine-root production by tree species and understorey functional groups in two contrasting peatland forests. *Plant Soil* 412 299–316.

[B4] Clemmensen K. E. Bahr A. Ovaskainen O. Dahlberg A. Ekblad A. Wallander H. (2013). Roots and associated fungi drive long-term carbon sequestration in boreal forest. *Science* 339 1615–1618. 10.1126/science.1231923 23539604

[B5] Dawson L. Mayes R. Elston D. Smart T. (2000). Root hydrocarbons as potential markers for determining species composition. *Plant, Cell Environ.* 23 743–750.

[B6] Domisch T. Finér L. Dawud S. M. Vesterdal L. Raulund-Rasmussen K. (2015). Does species richness affect fine root biomass and production in young forest plantations? *Oecologia* 177 581–594. 10.1007/s00442-014-3107-3 25300709

[B7] Duboc O. Zehetner F. Djukic I. Tatzber M. Berger T. Gerzabek M. (2012). Decomposition of European beech and Black pine foliar litter along an Alpine elevation gradient: mass loss and molecular characteristics. *Geoderma* 189 522–531. 10.1016/j.geoderma.2015.03.024 PMC4418737 26240437

[B8] Esbensen K. H. Guyot D. Westad F. Houmoller L. P. (2002). *Multivariate Data Analysis - in Practice: an Introduction to Multivariate Data Analysis and Experimental Design.* Oslo: CAMO Software.

[B9] Finér L. Domisch T. Dawud S. M. Raulund-Rasmussen K. Vesterdal L. Bouriaud O. (2017). Conifer proportion explains fine root biomass more than tree species diversity and site factors in major European forest types. *For. Ecol. Manage.* 406 330–350.

[B10] Finér L. Laine J. (1998). Root dynamics at drained peatland sites of different fertility in southern Finland. *Plant Soil* 201 27–36.

[B11] Finér L. Laine J. (2000). The ingrowth bag method in measuring root production on peatland sites. *Scand. J. For. Res.* 15 75–80.

[B12] Frolking S. Roulet N. T. Tuittila E. Bubier J. L. Quillet A. Talbot J. (2010). A new model of Holocene peatland net primary production, decomposition, water balance, and peat accumulation. *Earth System Dynamics* 1 1–21. 10.1111/gcb.12672

[B13] Frolking S. Talbot J. Jones M. C. Treat C. C. Kauffman J. B. Tuittila E. (2011). Peatlands in the Earth’s 21st century climate system. *Env. Rev.* 19 371–396.

[B14] Gill R. A. Jackson R. B. (2000). Global patterns of root turnover for terrestrial ecosystems. *New Phytol.* 147 13–31.

[B15] Gower S. Krankina O. Olson R. Apps M. Linder S. Wang C. (2001). Net primary production and carbon allocation patterns of boreal forest ecosystems. *Ecol. Appl.* 11 1395–1411.

[B16] Guo D. Xia M. Wei X. Chang W. Liu Y. Wang Z. (2008). Anatomical traits associated with absorption and mycorrhizal colonization are linked to root branch order in twenty-three Chinese temperate tree species. *New Phytol.* 180 673–683. 10.1111/j.1469-8137.2008.02573.x 18657210

[B17] Iversen C. M. Childs J. Norby R. J. Ontl T. A. Kolka R. K. Brice D. J. (2018). Fine-root growth in a forested bog is seasonally dynamic, but shallowly distributed in nutrient-poor peat. *Plant Soil* 424 123–143.

[B18] Jackson R. Canadell J. Ehleringer J. R. Mooney H. Sala O. Schulze E. (1996). A global analysis of root distributions for terrestrial biomes. *Oecologia* 108 389–411. 10.1007/BF00333714 28307854

[B19] Jandl R. Rodeghiero M. Martinez C. Cotrufo M. F. Bampa F. van Wesemael B. (2014). Current status, uncertainty and future needs in soil organic carbon monitoring. *Sci. Total Environ.* 468 376–383. 10.1016/j.scitotenv.2013.08.026 24041605

[B20] Joosten H. Clarke D. (2002). *Wise Use of Mires and Peatlands: Background and Principles Including a Framework for Decision-Making.* Saarijärvi: International Mire Conservation Group and International Peat Society.

[B21] Kaštovská E. Straková P. Edwards K. Urbanová Z. Bárta J. Mastný J. (2018). Cotton-grass and blueberry have opposite effect on peat characteristics and nutrient transformation in peatland. *Ecosystems* 21 443–458.

[B22] Kusumo B. H. Hedley M. J. Hedley C. B. Tuohy M. P. (2011). Measuring carbon dynamics in field soils using soil spectral reflectance: prediction of maize root density, soil organic carbon and nitrogen content. *Plant Soil* 338 233–245.

[B23] Laiho R. Bhuiyan R. Straková P. Mäkiranta P. Badorek T. Penttilä T. (2014). Modified ingrowth core method plus infrared calibration models for estimating fine root production in peatlands. *Plant Soil* 385 311–327.

[B24] Laiho R. Finér L. (1996). Changes in root biomass after water-level drawdown on pine mires in southern Finland. *Scand. J. For. Res.* 11 251–260.

[B25] Laiho R. Vasander H. Penttilä T. Laine J. (2003). Dynamics of plant-mediated organic matter and nutrient cycling following water-level drawdown in boreal peatlands. *Global Biogeochem. Cycles* 17:1053 10.1029/2002GB002015

[B26] Laine J. Vasander H. Laiho R. (1995). Long-term effects of water level drawdown on the vegetation of drained pine mires in southern Finland. *J. Appl. Ecol.* 32 785–802.

[B27] Legner N. Meinen C. Rauber R. (2018). Root differentiation of agricultural plant cultivars and proveniences using FTIR spectroscopy. *Front. Plant Sci.* 9:748 10.3389/fpls.2018.00748 PMC6008560 29951073

[B28] Lei P. Bauhus J. (2010). Use of near-infrared reflectance spectroscopy to predict species composition in tree fine-root mixtures. *Plant Soil* 333 93–103.

[B29] Lorenz K. Lal R. (2010). “Carbon dynamics and pools in major forest biomes of the world,” in *Carbon Sequestration in Forest Ecosystems* (Dordrecht: Springer), 159–205.

[B30] Meinen C. Rauber R. (2015). Root discrimination of closely related crop and weed species using FT MIR-ATR spectroscopy. *Front. Plant Sci.* 6:765 10.3389/fpls.2015.00765 PMC4586428 26483799

[B31] Minkkinen K. Ojanen P. Penttilä T. Aurela M. Laurila T. Tuovinen J. (2018). Persistent carbon sink at a boreal drained bog forest. *Biogeosciences* 15 3603–3624. 10.5194/bg-15-3603-2018

[B32] Mommer L. Dumbrell A. J. Wagemaker C. N. A. Ouborg N. J. (2011). Belowground DNA-based techniques: untangling the network of plant root interactions. *Plant Soil* 348 115–121. 10.1007/s11104-011-0962-0

[B33] Murphy M. Laiho R. Moore T. R. (2009a). Effects of water table drawdown on root production and aboveground biomass in a boreal bog. *Ecosystems* 12 1268–1282.

[B34] Murphy M. McKinley A. Moore T. (2009b). Variations in above-and below-ground vascular plant biomass and water table on a temperate ombrotrophic peatland. *Botany* 87 845–853.

[B35] Murphy M. T. Moore T. R. (2010). Linking root production to aboveground plant characteristics and water table in a temperate bog. *Plant Soil* 336 219–231.

[B36] Naumann A. Heine G. Rauber R. (2010). Efficient discrimination of oat and pea roots by cluster analysis of Fourier transform infrared (FTIR) spectra. *Field Crops Res.* 119 78–84.

[B37] Nichols J.E. Peteet D. M. (2019). Rapid expansion of northern peatlands and doubled estimate of carbon storage. *Nat. Geosci.* 12, 917–921. 10.1038/s41561-019-0454-z

[B38] Paavilainen E. Päivänen J. (1995). *Peatland Forestry: Ecology and Principles.* Berlin: Springer Science & Business Media.

[B39] Page S. E. Rieley J. O. Banks C. J. (2011). Global and regional importance of the tropical peatland carbon pool. *Global Change Biol.* 17 798–818.

[B40] Peltoniemi K. Straková P. Fritze H. Iráizoz P. A. Pennanen T. Laiho R. (2012). How water-level drawdown modifies litter-decomposing fungal and actinobacterial communities in boreal peatlands. *Soil Biol. Biochem.* 51 20–34.

[B41] Pena R. Lang C. Naumann A. Polle A. (2014). Ectomycorrhizal identification in environmental samples of tree roots by Fourier-transform infrared (FTIR) spectroscopy. *Front Plant Sci.* 5:229 10.3389/fpls.2014.00229 PMC4034152 24904624

[B42] Persson H. Å (1983). The distribution and productivity of fine roots in boreal forests. *Plant Soil* 71 87–101.

[B43] Picon-Cochard C. Pilon R. Revaillot S. Jestin M. Dawson L. (2009). Use of near-infrared reflectance spectroscopy to predict the percentage of dead versus living grass roots. *Plant Soil* 317 309–320.

[B44] Pregitzer K. S. DeForest J. L. Burton A. J. Allen M. F. Ruess R. W. Hendrick R. L. (2002). Fine root architecture of nine North American trees. *Ecol. Monogr.* 72 293–309.

[B45] Proctor C. He Y. (2019). Quantifying wetland plant vertical root distribution for estimating the Interface with the anoxic zone. *Plant Soil* 440 381–398.

[B46] Rewald B. Meinen C. (2013). Plant roots and spectroscopic methods–analyzing species, biomass and vitality. *Front Plant Sci.* 4:393 10.3389/fpls.2013.00393 PMC3793172 24130565

[B47] Robroek B. J. Jassey V. E. Kox M. A. Berendsen R. L. Mills R. T. Cécillon L. (2015). Peatland vascular plant functional types affect methane dynamics by altering microbial community structure. *J. Ecol.* 103 925–934.

[B48] Roumet C. Picon-Cochard C. Dawson L. A. Joffre R. Mayes R. Blanchard A. (2006). Quantifying species composition in root mixtures using two methods: near-infrared reflectance spectroscopy and plant wax markers. *New Phytol.* 170 631–638. 10.1111/j.1469-8137.2006.01698.x 16626482

[B49] Rumbaugh M. Clark D. Pendery B. (1988). Determination of root mass ratios in alfalfa-grass mixtures using near infrared reflectance spectroscopy. *J. Range Manag.* 41 488–490.

[B50] Rydin H. Jeglum J. K. (2013). *The Biology of Peatlands.* Oxford: Oxford university press.

[B51] Saarinen T. (1996). Biomass and production of two vascular plants in a boreal mesotrophic fen. *Can. J. Bot.* 74 934–938.

[B52] Sjörs H. (1991). Phyto-and necromass above and below ground in a fen. *Ecography* 14 208–218.

[B53] Straková P. Anttila J. Spetz P. Kitunen V. Tapanila T. Laiho R. (2010). Litter quality and its response to water level drawdown in boreal peatlands at plant species and community level. *Plant Soil* 335 501–520.

[B54] Straková P. Niemi M. Freeman C. Peltoniemi K. Toberman H. Heiskanen I. (2011). Litter type affects the activity of aerobic decomposers in a boreal peatland more than site nutrient and water table regimes. *Biogeosciences* 8, 2741–2755. 10.5194/bg-8-2741-2011

[B55] Straková P. Penttilä T. Laine J. Laiho R. (2012). Disentangling direct and indirect effects of water table drawdown on above-and belowground plant litter decomposition: consequences for accumulation of organic matter in boreal peatlands. *Global Change Biol.* 18 322–335.

[B56] Streit J. Meinen C. Nelson W. C. D. Siebrecht-Schöll D. J. Rauber R. (2019). Above-and belowground biomass in a mixed cropping system with eight novel winter faba bean genotypes and winter wheat using FTIR spectroscopy for root species discrimination. *Plant Soil* 436 141–158.

[B57] Thomas F. M. Molitor F. Werner W. (2014). Lignin and cellulose concentrations in roots of Douglas fir and European beech of different diameter classes and soil depths. *Trees* 28 309–315.

[B58] Tong J. Xiang W. Lei P. Liu J. Tian D. Deng X. (2016). Prediction of tree species composition in fine root mixed samples using near-infrared reflectance spectroscopy. *Plant Biosyst.* 150 412–419.

[B59] Vávřová P. Penttilä T. Laiho R. (2009). Decomposition of Scots pine fine woody debris in boreal conditions: Implications for estimating carbon pools and fluxes. *For. Ecol. Manag.* 257 401–412.

[B60] Weishampel P. Kolka R. King J. Y. (2009). Carbon pools and productivity in a 1-km2 heterogeneous forest and peatland mosaic in Minnesota. *USA. For. Ecol. Manag.* 257 747–754.

[B61] Westoby M. Wright I. J. (2006). Land-plant ecology on the basis of functional traits. *Trends Ecol. Evol.* 21 261–268. 10.1016/j.tree.2006.02.004 16697912

[B62] White K. E. Reeves J. B. III Coale F. J. (2011). Mid-infrared diffuse reflectance spectroscopy for the rapid analysis of plant root composition. *Geoderma* 167 197–203.

[B63] Zhang C. Chen L. Jiang J. (2014). Why fine tree roots are stronger than thicker roots: the role of cellulose and lignin in relation to slope stability. *Geomorphology* 206 196–202.

