# Supplementary material for: Quantification of Plant Root Species Composition in Peatlands Using FTIR Spectroscopy
Source: Front Plant Sci. 2020 May 19;11:597. doi: 10.3389/fpls.2020.00597 (PMC7250167; doi:10.3389/fpls.2020.00597)
Supplement: Supplementary file 1 [file Data_Sheet_1.pdf]

Supplementary Material

1 Collection and organization of the root materials into sample sets

The root materials for *calibration sample sets I and II* were collected in open (Mäkiranta et al., 2018) and forested (Bhuiyan et al., 2017) peatland sites in Finland. The roots were collected by extracting whole plants with their root system for shrubs and herbaceous plants, or by removing the surface peat and following several roots from the trunk base till the root ends for trees, these root systems then being harvested. The plants/roots were transported to a laboratory where the roots were cut and rinsed with water to clean out the attached soil. Shrub and tree roots were sorted to fine (diameter < 2 mm) and coarser (diameter 2−10 mm) roots.

The *calibration sample sets I and II* included two types of dead roots, field-dead roots that were collected from living plants (*Carex rostrata*, *Eriophorum vaginatum*, *Pinus sylvestris* and *Vaccinium myrtillus*) in the field and separated from living roots using morphological criteria (colour, structure and strength), as well as artificially-dead roots of *Pinus sylvestris* and *Vaccinium myrtillus* produced in a root mortality treatment where root death was induced by desiccation, as in Nakaji et al. (2008). The *Pinus sylvestris* plants used for producing the artificially-dead roots were originally 1-year seedlings from a tree nursery, while the *Vaccinium myrtillus* plants were collected in the field. Both species were then planted in pure *Sphagnum* peat for three weeks to produce roots and then artificially killed by drought (stop of water supply) in a greenhouse. For being eventually dead, *Vaccinium myrtillus* was kept without water supply for 8 weeks, while *Pinus sylvestris* for 11 weeks in combination with shading for the last 3 weeks. The aim of the artificial killing was to obtain dead, but still largely undecomposed roots. Differently from Nakaji et al. (2008) and Picon-Cochard et al. (2009), aboveground plant parts were not cut to allow potential translocation from the dying roots.

For *external validation sample sets III and IV*, living herbaceous and woody roots were collected in open (Mäkiranta et al., 2018) and forested (Bhuiyan et al., 2017) peatland sites in Finland (woody roots at one site only) in the same way as described above for *calibration sample sets I and II*, but in different years.

For *external validation sample set V*, very fine roots of woody plants of diameter ≤ 0.5 mm were extracted from selected stored samples of *II* and *IV*, and newly collected for *Andromeda polifolia*, *Betula nana* and *Vaccinium oxycoccos* at two open peatland sites (Mäkiranta et al., 2018) in Finland by extracting whole plants with their root system.

The *additional mixtures sample set VI* consist of mixtures that were prepared using roots of *external validation sample sets III* and *V*.

Distant validation sample set VII included roots of graminoid *Eriophorum vaginatum* and very fine (diameter ≤ 0.5 mm) roots of five shrub species that were collected at peatland sites in Canada (Murphy et al., 2010), Sweden (Nilsson et al., 2008) and UK (Levy et al., 2018), by removing the surface peat and following several roots from the plant base till the root ends. The *Eriophorum vaginatum* and *Vaccinium myrtillus* roots from a spruce swamp forest site in the Czech Republic (Kaštovská et al., 2018) were collected under monospecific patches of the given species and included both living and dead roots. The live and dead roots were hand-sorted in a laboratory and determined visually by colour and firmness. The roots that were identified dead represented field-dead roots in this study.

All processed root samples were dried at 25−30 °C or freeze-dried, except for the roots from the Czech Republic in the *distant validation sample set VII* that were dried at 60 °C for 72 h. After drying, the samples were stored in paper bags or plastic tubes in dry conditions at a room temperature (21−22 °C).

2 Model validation: internal, external, distant

When comparing performances of different types of models or models from different studies, it is necessary to be aware of the procedure that was used for model validation to make safe interpretations (Bellon-Maurel and McBratney, 2011). Internal leave-one-out cross-validation (e.g., Domisch et al., 2015; Meinen and Rauber, 2015; Tong et al., 2016; Finér et al., 2017) always gives the best results. In published root studies this is due to the fact that the samples themselves were not fully independent, but for example came from narrow sampling and represented subsamples prepared from the same initial bulk root samples (e.g., Meinen and Rauber, 2015; Tong et al., 2016; Streit et al., 2019). In such case the validation samples have same origin as samples of the calibration set. However, leave-one-out cross validation is rigorously independent if the samples themselves are highly independent (Bellon-Maurel and McBratney, 2011).

When an “external” validation set is built only by dividing the original sample set into two parts, one for calibration and one for validation, as in e.g., Lei and Bauhus (2010); Meinen and Rauber (2015); Streit et al. (2019), performance is altered compared to the leave-one-out cross-validation, simply because the calibration sample number is reduced (Bellon-Maurel and McBratney, 2011). Whether this way of validation can be considered as independent, again bank on the fact whether the samples themselves within the original dataset were fully independent.

Performance of the calibration models always drops when applied on really independent validation samples, which is however what is most likely to be encountered in reality (Bellon-Maurel and McBratney, 2011). In case of roots this may come from the fact that the independent samples are influenced by different parameters than were the calibration samples, such as different temperature, water supply, nutrition, mycorrhizal colonization, etc., which may affect root chemistry and thus FTIR signatures but are not fully related to the target variable which is plant species or root type.

We used leave-one-out cross validation when building our calibration models to use maximum available samples for the model calibration. We also tested the way of validation by dividing the calibration sample set into two parts, one for calibration (75% of the sample set; every first, second and third sample) and one for validation (25% of the sample set; every fourth sample). The validation outcomes were comparable to the outcomes obtained with the full cross validation (data not shown) and thus we rather selected the full cross validation as it enabled using maximum number of available samples for building the calibration models. Additional external and distant validation on fully independent local and foreign sample sets from Finland, Canada, Czech Republic, Sweden and UK (Table 1, Figure 1) was used to test the real applicability of the models in forthcoming studies.

2.1 Root type level models

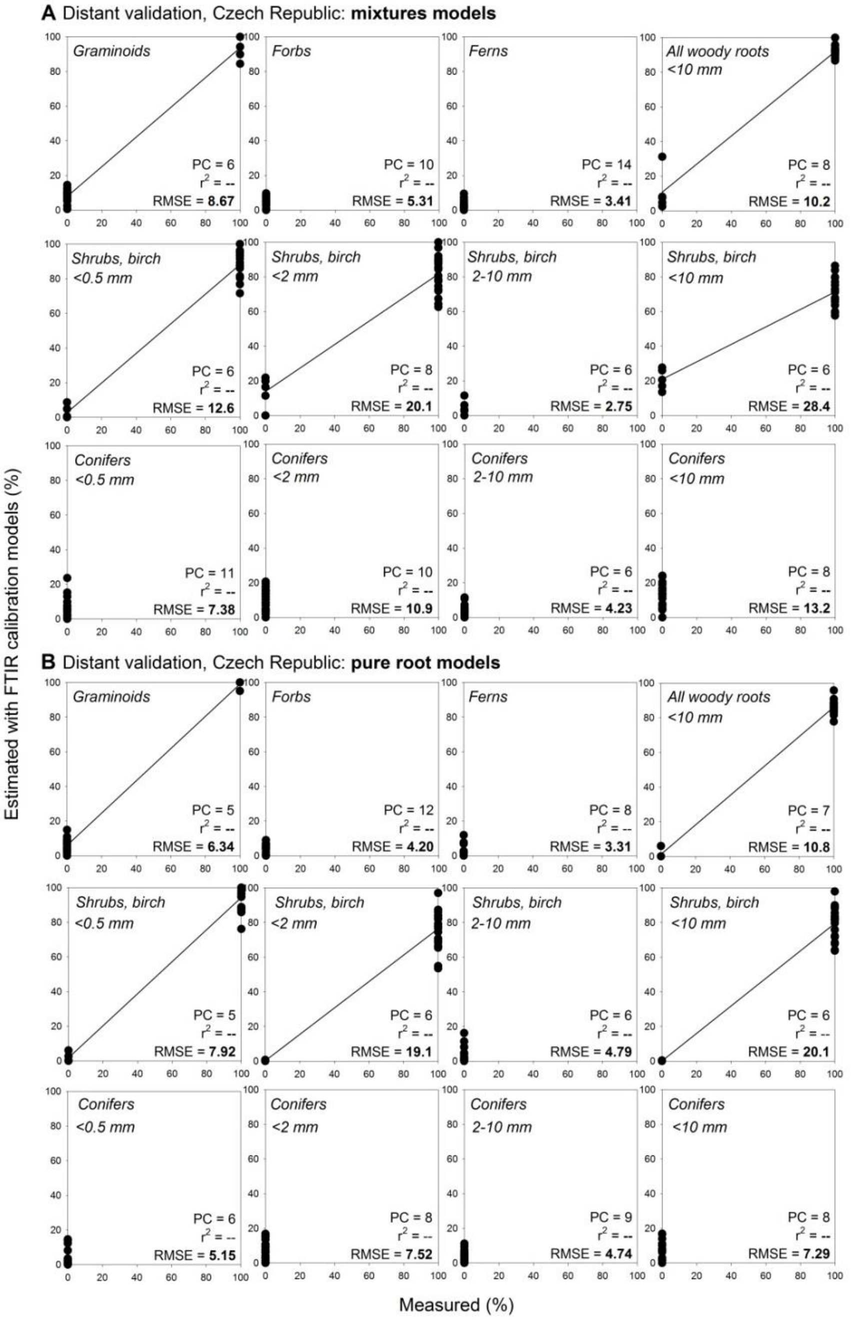

Supplementary Figure S1| Distant validation, Czech Republic, of the root type level calibration models: comparison of estimates using mixtures models and pure roots models.
Relationships between the measured percentage of roots of the specific root type in composite root samples and the percentage estimated using FTIR calibration models: comparison of estimates using (A) mixtures models and (B) pure roots models. The calibration models are presented in Figure 6. Distant validation of the models show samples from spruce swamp forest site in the Czech Republic (*distant validation sample set VII*) that included **living roots** of plant species present in the calibration (living roots of *Vaccinium myrtillus* collected in May were removed as outliers), n = 25. PC is the number of factors ("principal components") included in the calibration models and RMSE is the root mean square error of the prediction.

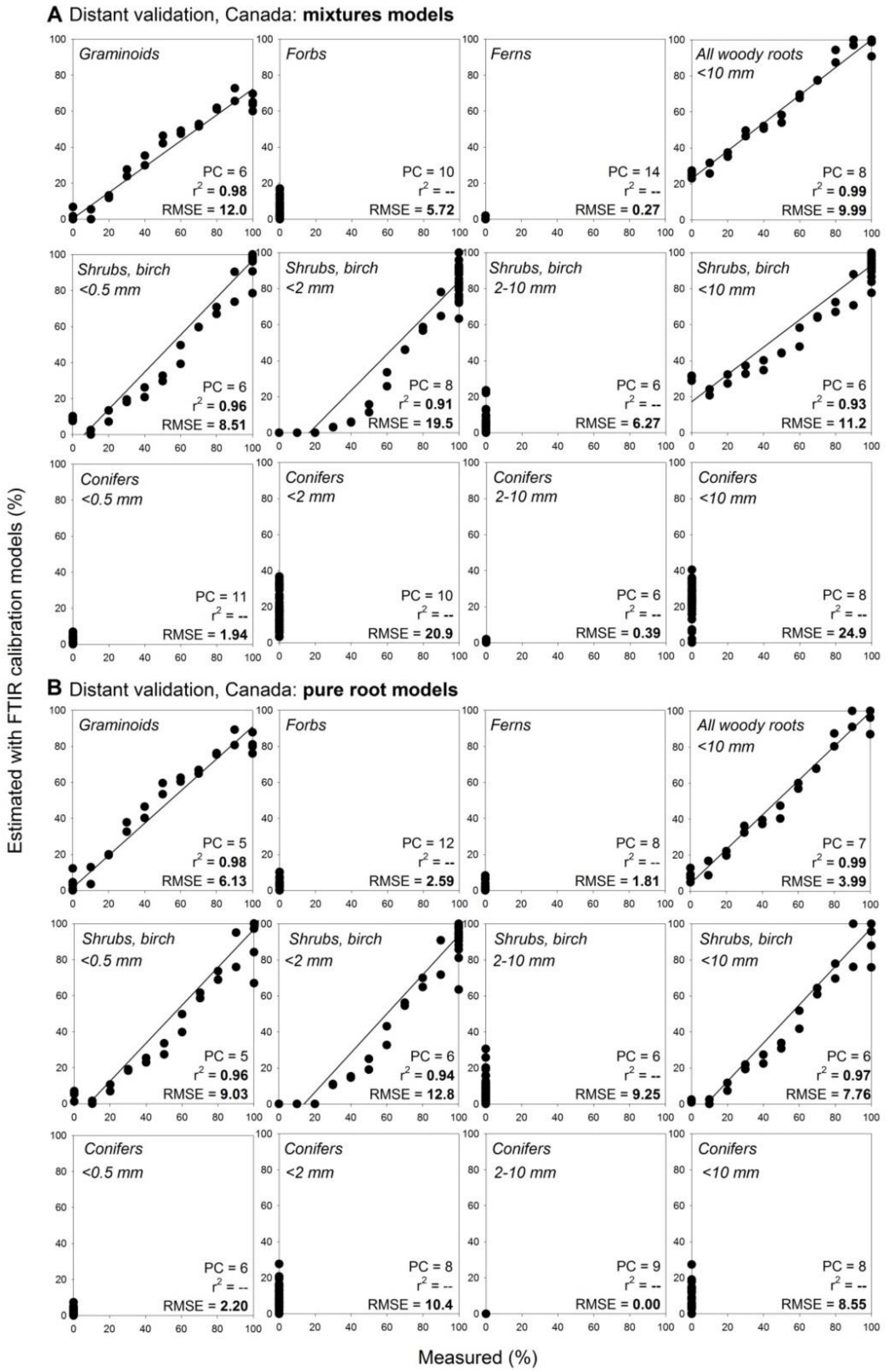

Supplementary Figure S2| Distant validation, Canada, of the root type level calibration models: comparison of estimates using mixtures models and pure roots models.

Relationships between the measured percentage of roots of the specific root type in composite root samples and the percentage estimated using FTIR calibration models: comparison of estimates using (A) mixtures models and (B) pure roots models. The calibration models are presented in Figure 6. Distant validation of the models show samples from bog site in Canada (*distant validation sample set VII*) that included roots of different shrub species than those present in the calibration, n = 57. PC is the number of factors ("principal components") included in the calibration models and RMSE is the root mean square error of the prediction.

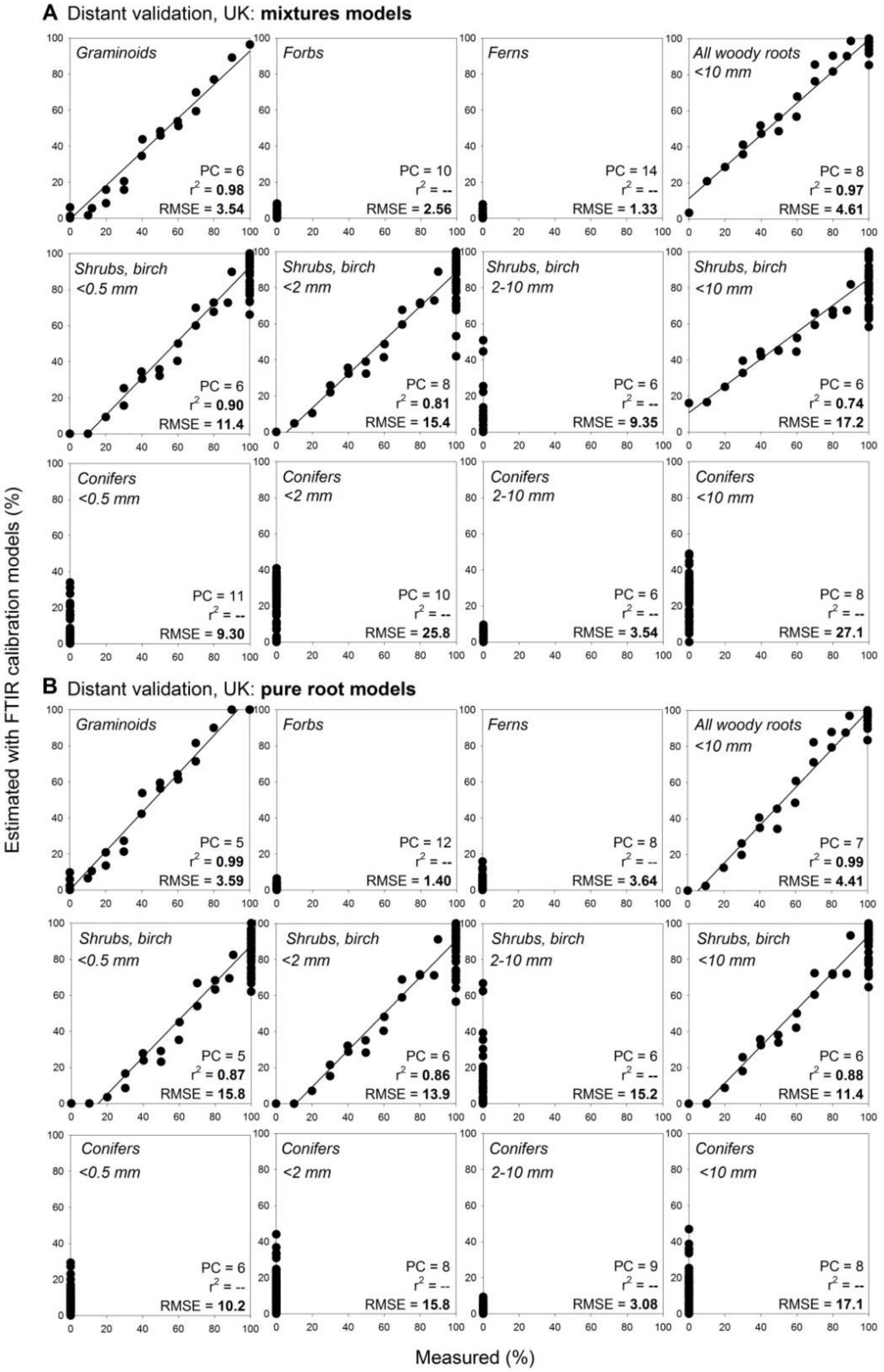

Supplementary Figure S3| Distant validation, UK, of the root type level calibration models: comparison of estimates using mixtures models and pure roots models.

Relationships between the measured percentage of roots of the specific root type in composite root samples and the percentage estimated using FTIR calibration models: comparison of estimates using (A) mixtures models and (B) pure roots models. The calibration models are presented in Figure 6. Distant validation of the models show samples from bog site in UK (*distant validation sample set VII*) that included roots of different shrub species than those present in the calibration, n = 73. PC is the number of factors ("principal components") included in the calibration models and RMSE is the root mean square error of the prediction.

2.2 Species level models

2.2.1 Herbaceous species

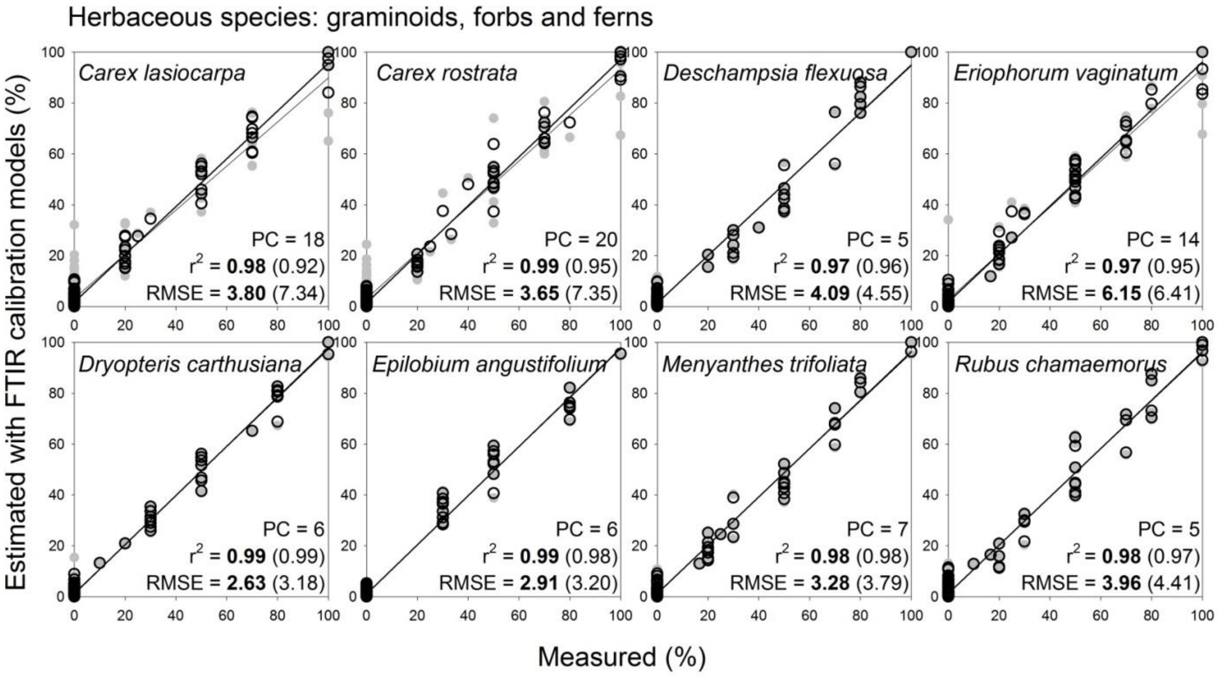

Supplementary Figure S4| Species level models for herbaceous species and their internal validation.
Relationships between the measured percentage of roots of the specific herbaceous plant species in composite root samples and the percentage estimated using FTIR. For calibration, samples from *calibration sample set I* were used, n = 127. PC is the number of factors (“principal components”) included in the calibration models and RMSE is the root mean square error of the prediction. Calibration values are shown by black open symbols with RMSE and r^2 in bold letters, the internal full-cross validation values are shown by grey symbols with values of RMSE and r^2 in parentheses.

2.2.2 Woody species

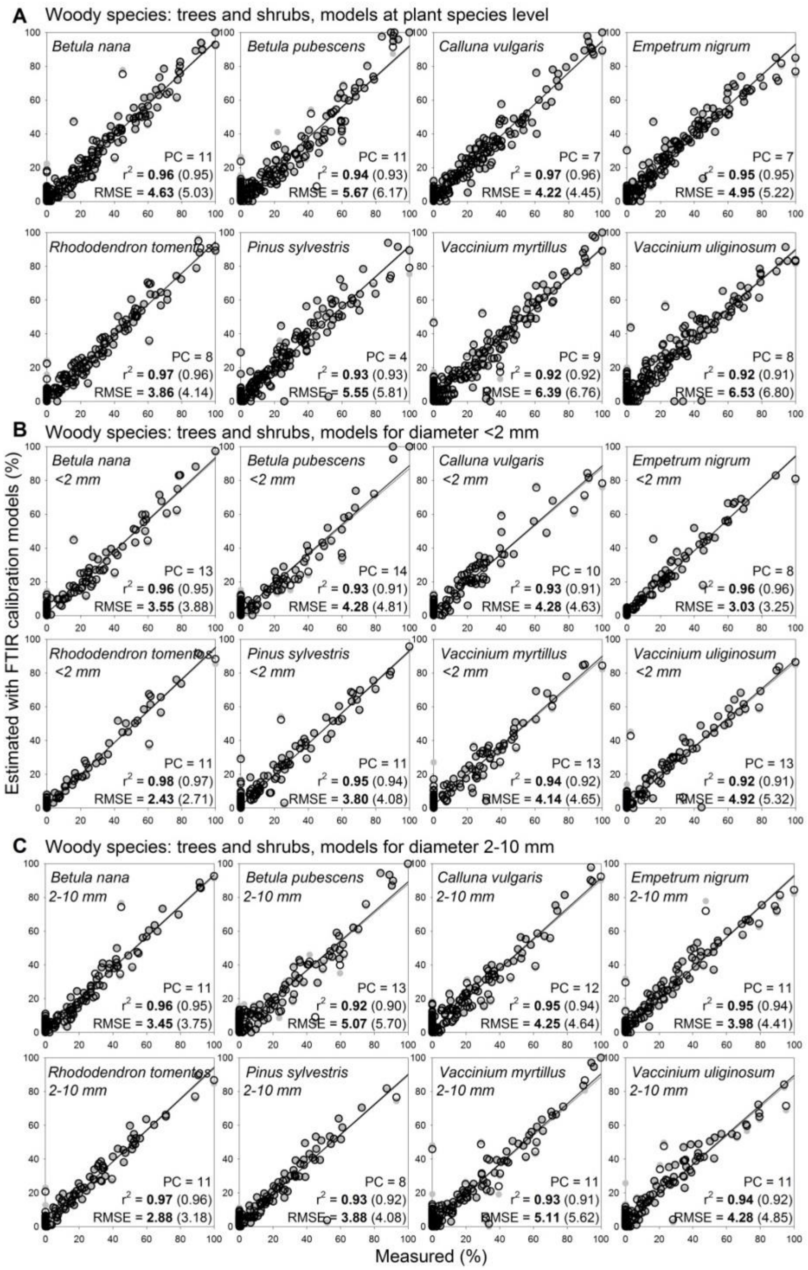

Supplementary Figure S5| Species level calibration models (narrow) for woody roots and their internal validation, with separate diameter class level calibration models for fine (diameter < 2 mm) and coarser (diameter 2–10 mm) roots.

Relationships between the measured percentage of roots of the specific woody plant species in composite root samples and the percentage estimated using FTIR. For calibration, samples from *external validation sample set IV* were used, n = 367. The graphs represent (A) species level calibration models, (B) calibration models for fine roots (diameter < 2 mm), (C) calibration models for coarser roots (diameter 2–10 mm). PC is the number of factors (“principal components”) included in the calibration models and RMSE is the root mean square error of the prediction. Calibration values are shown by black open symbols with RMSE and r^2 in bold letters, the internal full-cross validation values are shown by grey symbols with values of RMSE and r^2 in parentheses.

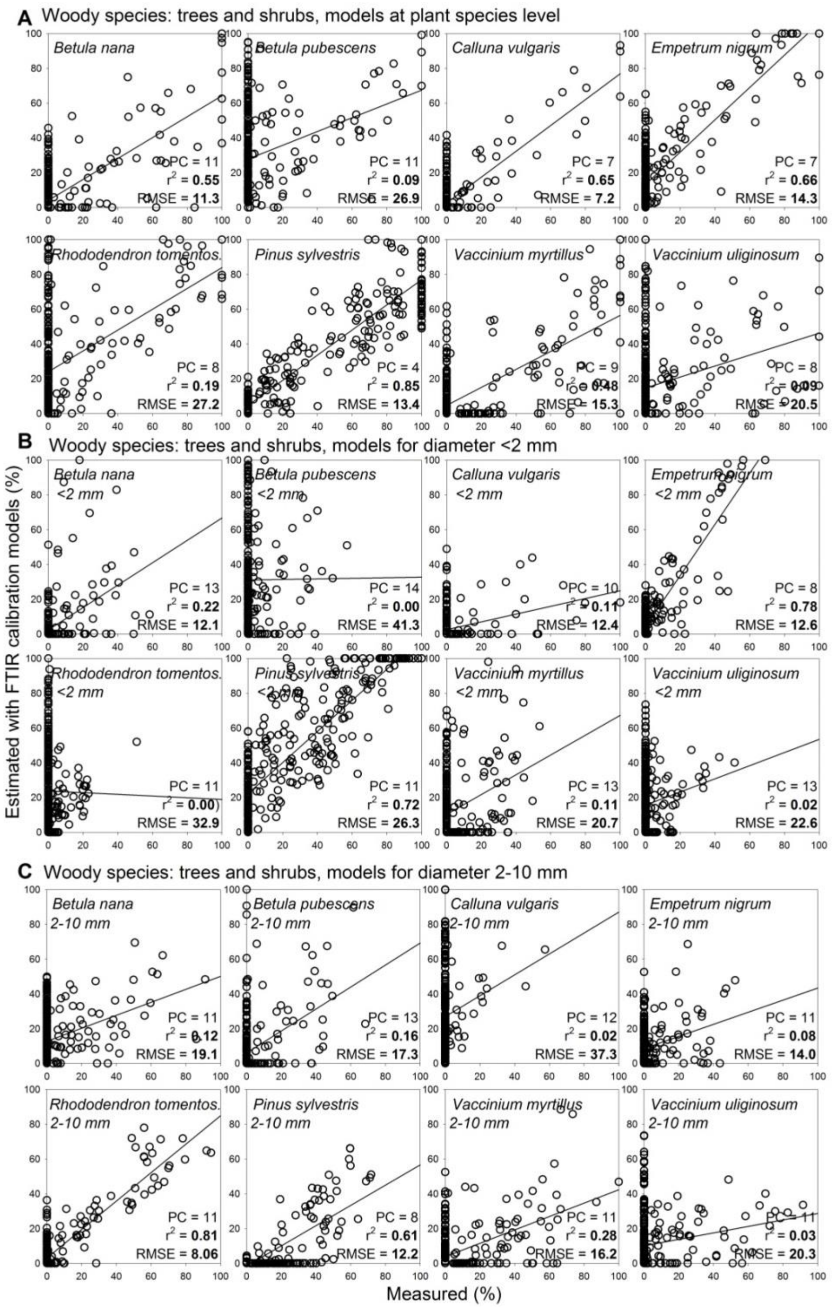

Supplementary Figure S6| External validation of the narrow species and diameter class level calibration models for woody roots.

Relationships between the measured percentage of roots of the specific woody plant species in composite root samples and the percentage estimated using FTIR calibration models, the calibration models are presented in Figure S5. For external validation of the calibration models, samples from *external validation sample set II* were used, but samples that contained *Picea abies* and *Vaccinium vitis-idaea* (species not present in the calibration models) were filtered out, n = 294. The graphs represent estimates using (A) species level calibration models, (B) calibration models for fine roots (diameter < 2 mm), (C) calibration models for coarser roots (diameter 2–10 mm). PC is the number of factors (“principal components”) included in the calibration models and RMSE is the root mean square error of the prediction.

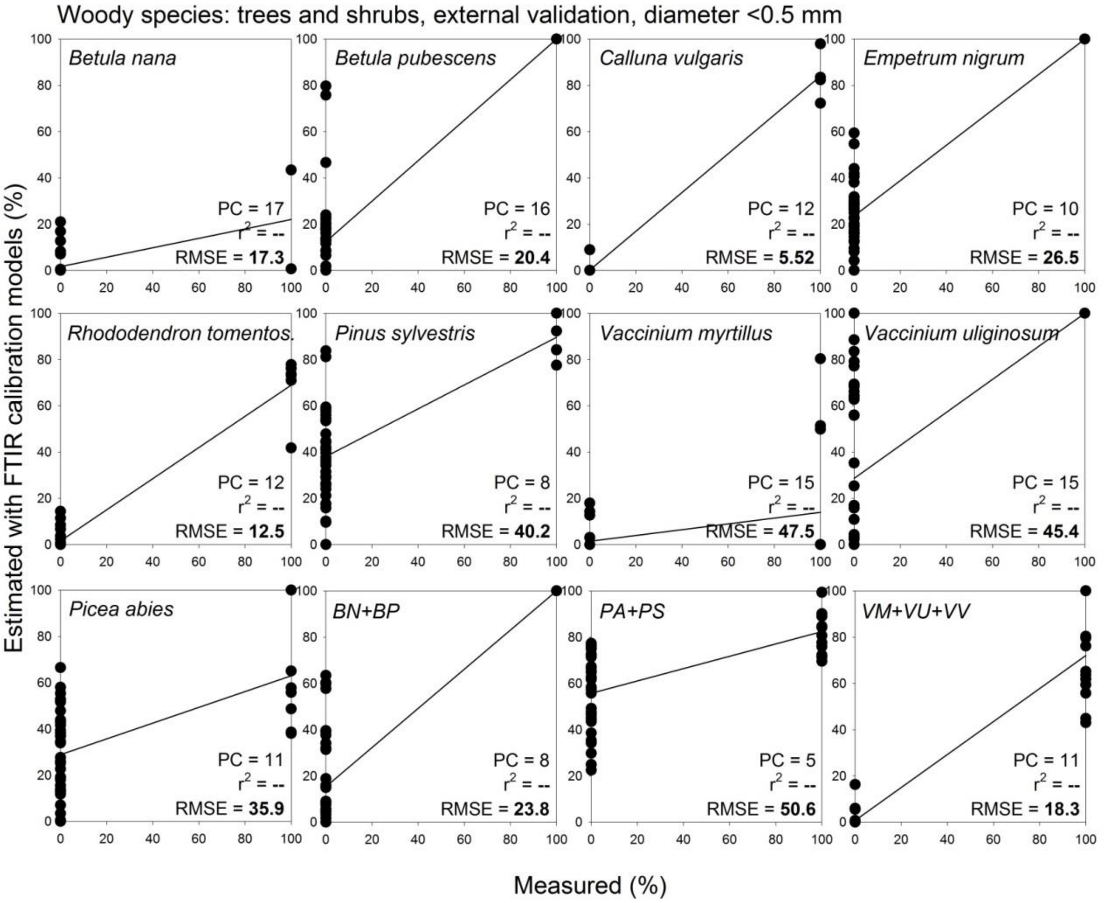

Supplementary Figure S7| External validation of the broader species level calibration models for woody roots on roots of the same species but very fine diameter (≤ 0.5 mm). Relationships between the measured percentage of roots of the specific woody plant species in composite root samples and the percentage estimated using FTIR calibration models, the calibration models are presented in Figure 8A. For external validation of the calibration models, samples from *external validation sample set V* were used, except for *Andromeda polifolia* and *Vaccinium oxycoccos* (species not present in the calibration models), n = 52. PC is the number of factors ("principal components") included in the calibration models and RMSE is the root mean square error of the prediction. Species abbreviations: BN+BP, *Betula nana* with *B. pubescens;* PA+PS, *Picea abies* with *Pinus sylvestris*; VM+VU+VV, *Vaccinium myrtillus* with *V. uliginosum* and *V. vitis-idaea*.

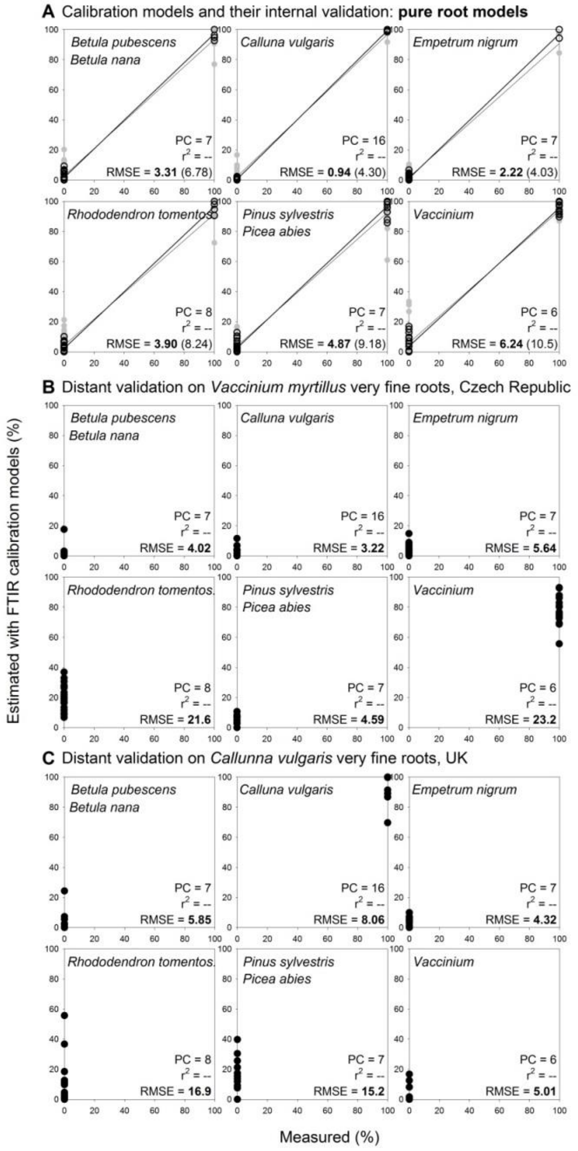

Supplementary Figure S8| Species level calibration models for very fine (diameter ≤ 0.5 mm) woody roots and their distant validation.

Relationships between the measured percentage of roots of the specific woody plant species in composite root samples and the percentage estimated using FTIR calibration models.

(A) Calibration models and their internal validation. For calibration, pure root samples from *external validation sample set V* (excluding open peatland species *Andromeda polifolia* and *Vaccinium oxycoccos*) were used, n=52. (B) Distant validation of the calibration models on *Vaccinium myrtillus* living very fine roots from the site in the Czech Republic (*distant validation sample set VII*), living roots collected in May were removed as outliers, n=25. (C) Distant validation of the calibration models on *Calluna vulgaris* very fine roots from the site in UK (*distant validation sample set VII*), n=20. PC is the number of factors ("principal components") included in the calibration models and RMSE is the root mean square error of the prediction. Calibration values are shown by black open symbols with RMSE and r^2 in bold letters, the internal full-cross validation values are shown by grey symbols with values of RMSE and r^2 in parentheses, and the distant validation values are shown by black full symbols. Vaccinium is *Vaccinium myrtillus* with *V. uliginosum* and *V. vitis-idaea*.

2.2.3 General models (herbaceous and woody species together)

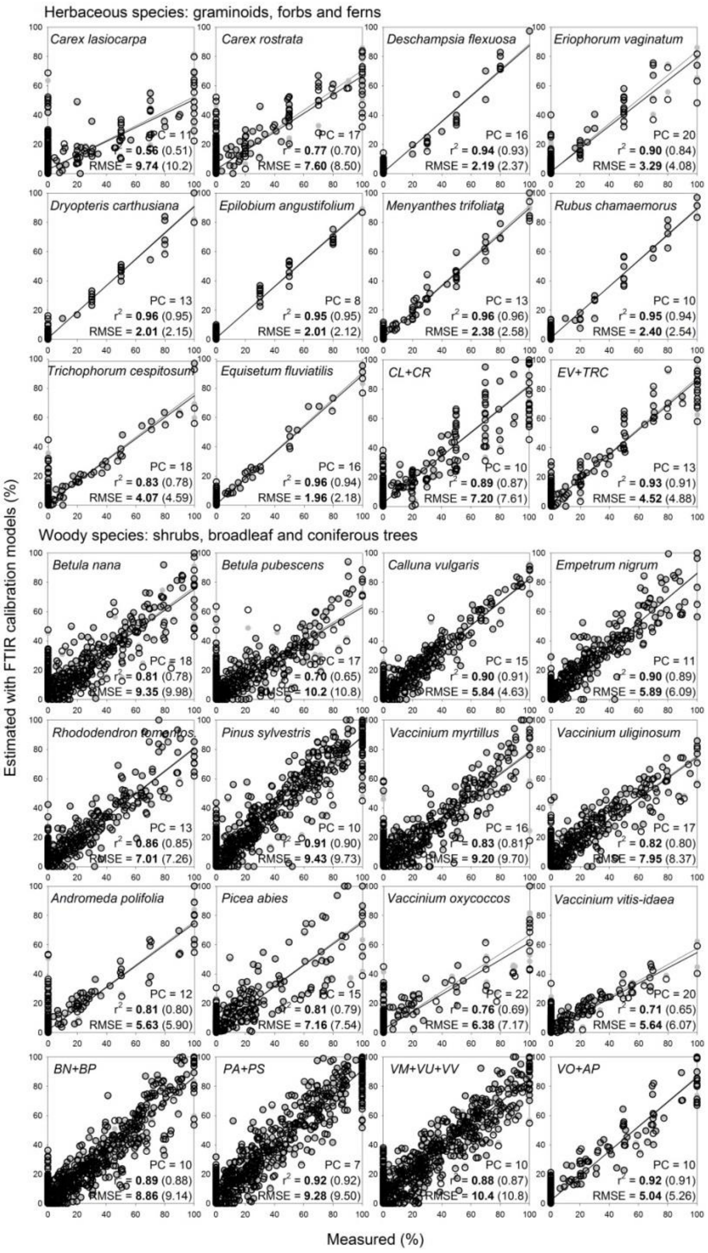

Supplementary Figure S9| General species level calibration models for herbaceous and woody roots and their internal validation.

Relationships between the measured percentage of roots of the specific (A) herbaceous and (B) woody plant species in composite root samples and the percentage estimated using FTIR calibration models. For calibration, samples from *calibration* and *external validation sample sets I–IV*, *VI* (Table 1, Figure 1) were used, n = 1166. PC is the number of factors ("principal components") included in the calibration models and RMSE is the root mean square error of the prediction. Calibration values are shown by black open symbols with RMSE and r^2 in bold letters, the internal full-cross validation values are shown by grey symbols with values of RMSE and r^2 in parentheses. Species abbreviations: BN+BP, *Betula nana* with *B. pubescens;* PA+PS, *Picea abies* with *Pinus sylvestris*; VM+VU+VV, *Vaccinium myrtillus* with *V. uliginosum* and *V. vitis-idaea*; VO+AP, *Vaccinium oxycoccos* with *Andromeda polyfolia*.

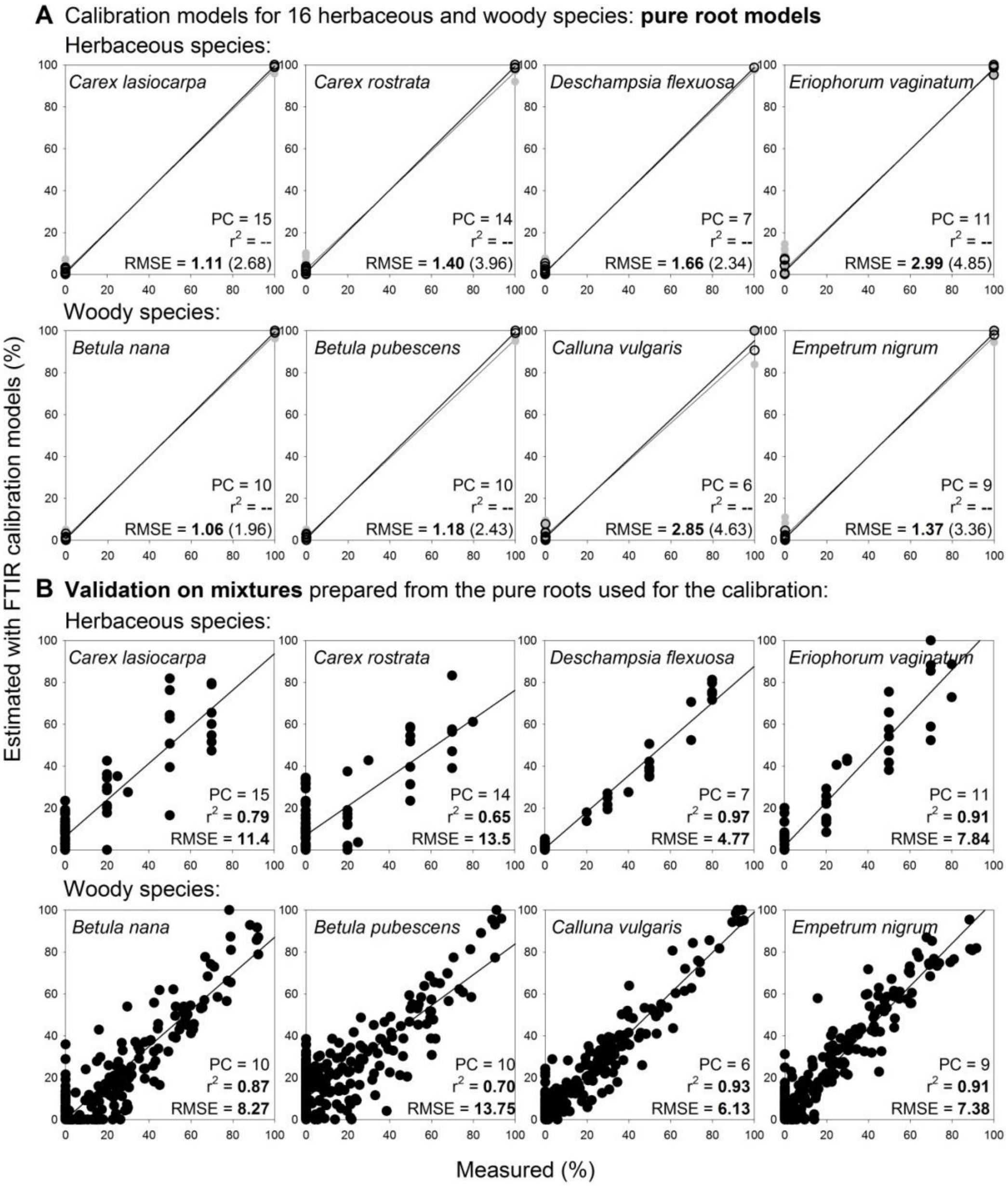

Supplementary Figure S10| Species level calibration models constructed on pure roots of 16 species and their validation on mixtures prepared from the pure roots used for the calibration. Relationships between the measured percentage of roots of the different plant species in composite root samples and the percentage estimated with FTIR calibration models.(A) For calibration, pure roots of 16 herbaceous and woody species of the *calibration* and *external validation sample sets I* and *IV* were used, only living roots were included, n = 38. (B) Validation of the models show artificial mixtures containing known mass proportions of roots of the given species, that were prepared from the pure roots used for the calibration, n = 466. PC is the number of factors ("principal components") included in the calibration models and RMSE is the root mean square error of the prediction. Calibration values are shown by black open symbols with RMSE and r^2 in bold letters, the internal full-cross validation values are shown by grey symbols with values of RMSE and r^2 in parentheses, and the distant validation values are shown by black full symbols. Only graphs for 8 species are shown, graphs for the remaining 8 species are shown in Figure 10.

3 Dead roots

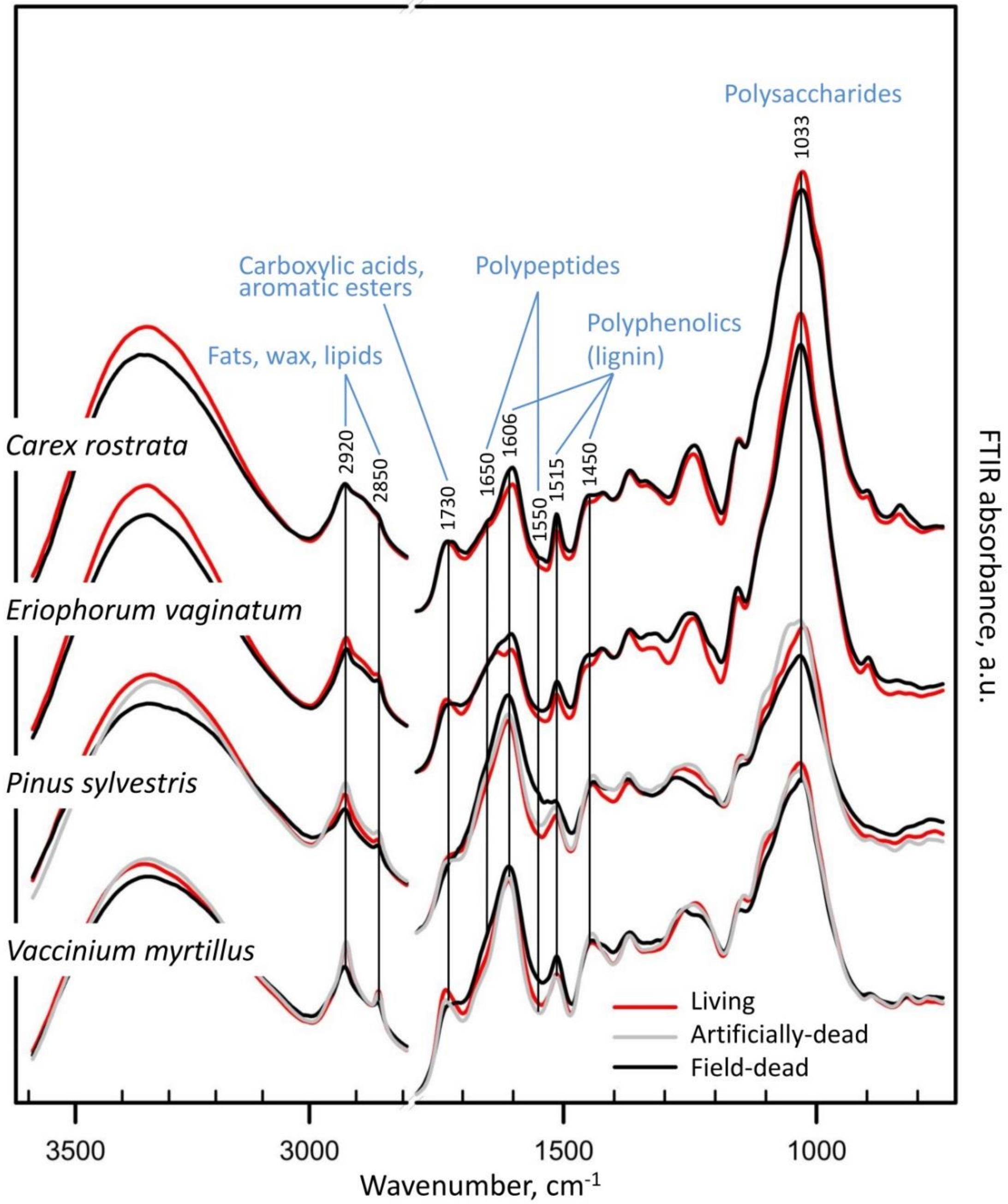

Supplementary Figure S11| Comparison of FTIR spectra of living and dead roots.
FTIR absorbance spectra of dead and living roots of four plant species from the *calibration* and *external validation datasets I* and *III* (Table 1, Figure 1). The lines represent mean spectra for each species and living or dead root variant.

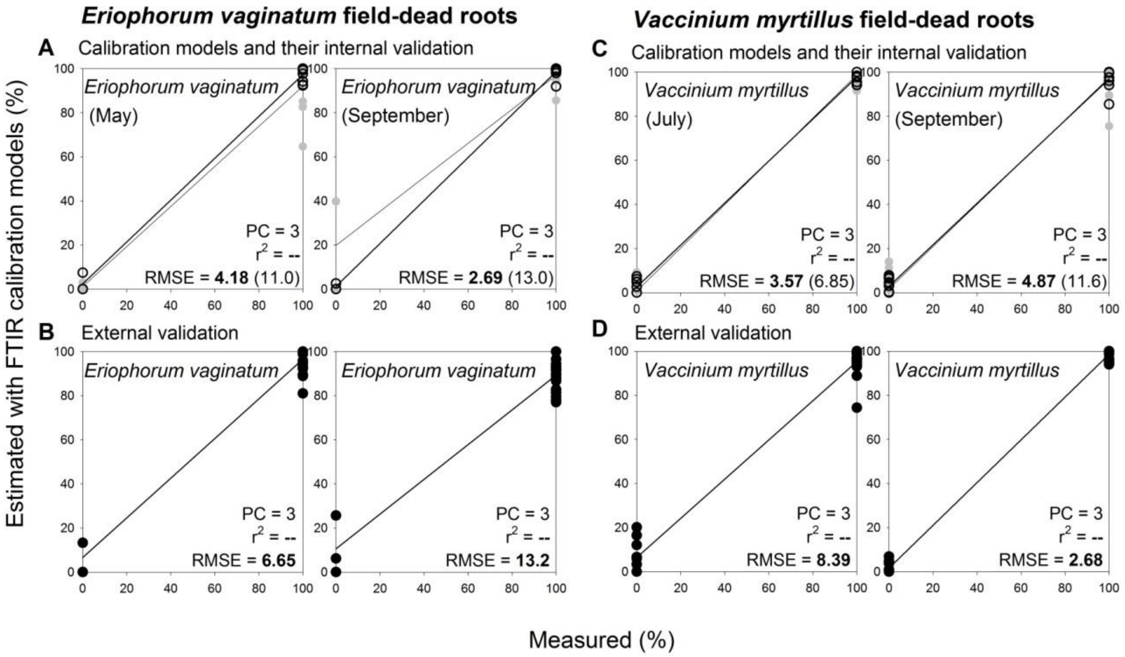

Supplementary Figure S12| Calibration models for dead root quantification within the given species, and their external validation.

Relationships between the measured percentage of dead roots of the given plant species and the percentage estimated with FTIR calibration models. (A) For calibration, living and field-dead pure roots of *Eriophorum vaginatum* from spruce swamp forest site in the Czech Republic (*distant validation sample set VII*) were used, and separate models were constructed for the roots collected at two different times: May or September. (B) External validation of the *Eriophorum vaginatum* models was performed using samples of the same site but collected at different times than the roots used in the calibration models: July and September roots for the May roots model, July and May roots for the September roots model. (C) For calibration, living and field-dead pure roots of *Vaccinium myrtillus* from spruce swamp forest site in the Czech Republic (*distant validation sample set VII*) were used, and separate models were constructed for the roots collected at two different times: July or September. (D) External validation of the *Vaccinium myrtillus* models was performed using samples of the same site but collected at different times than the roots used in the calibration models: May (only dead, living were excluded as outliers) and September roots for the July roots model, May (only dead, living were excluded as outliers) and July for the September roots model. PC is the number of factors ("principal components") included in the calibration models and RMSE is the root mean square error of the prediction. Calibration values are shown by black open symbols with RMSE and r^2 in bold letters, the internal full-cross validation values are shown by grey symbols with values of RMSE and r^2 in parentheses, and the external validation values are shown by black full symbols.

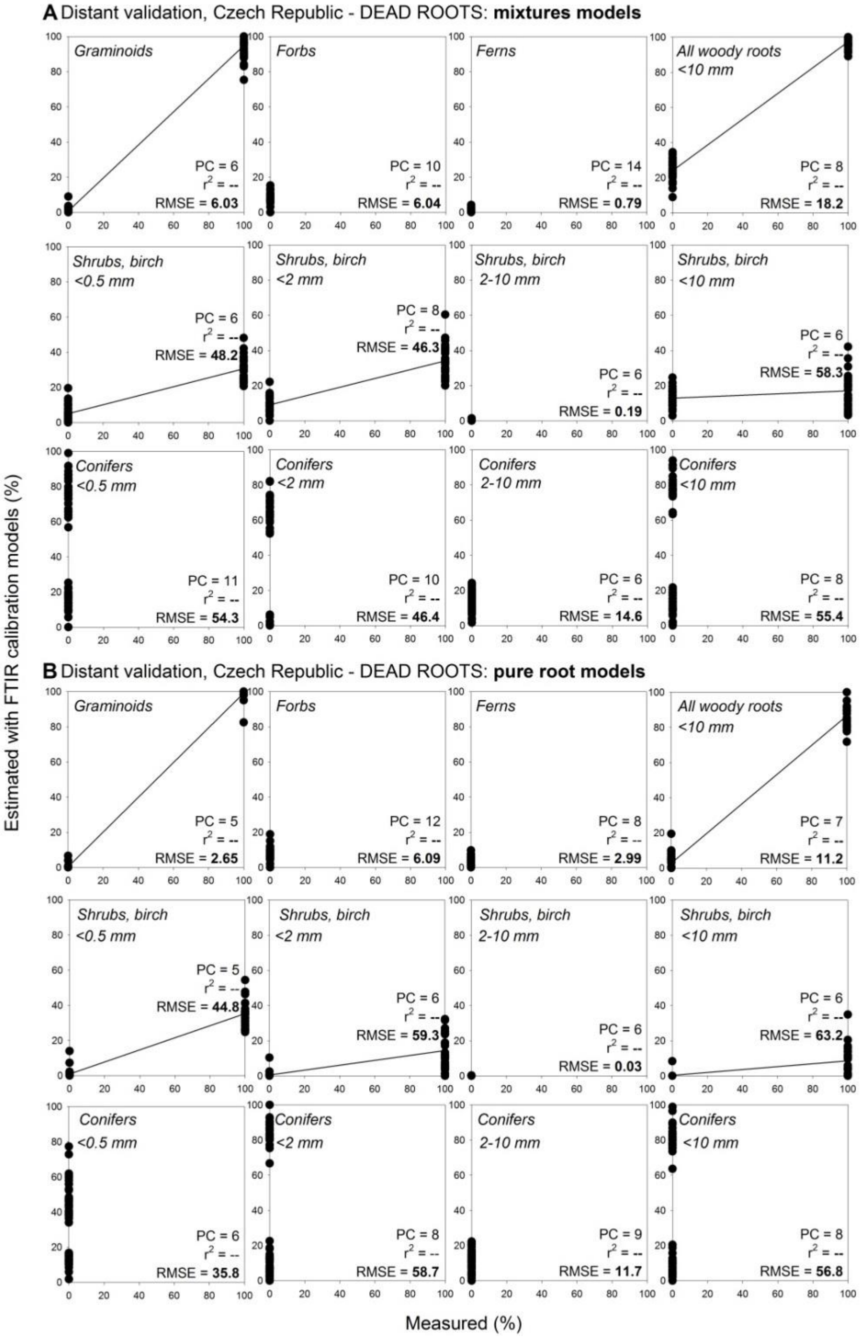

Supplementary Figure S13| Distant validation of the root type level calibration models on field-dead roots of *Eriophorum vaginatum* and *Vaccinium myrtillus*, Czech Republic: comparison of estimates using mixtures models and pure roots models.

Relationships between the measured percentage of roots of the specific root type in composite root samples and the percentage estimated using FTIR calibration models: comparison of estimates using (A) mixtures models and (B) pure roots models. The calibration models are presented in Figure 6. Distant validation of the models show samples from spruce swamp forest site in the Czech Republic (*distant validation sample set VII*) that included **dead roots** of plant species present in the calibration, n = 57. PC is the number of factors ("principal components") included in the calibration models and RMSE is the root mean square error of the prediction.

4 References

Bellon-Maurel, V. and McBratney, A. (2011). Near-infrared (NIR) and mid-infrared (MIR) spectroscopic techniques for assessing the amount of carbon stock in soils–Critical review and research perspectives. *Soil Biol. Biochem.* 43, 1398–1410.

Bhuiyan, R., Minkkinen, K., Helmisaari, H., Ojanen, P., Penttilä, T., and Laiho, R. (2017). Estimating fine-root production by tree species and understorey functional groups in two contrasting peatland forests. *Plant Soil* 412, 299–316.

Domisch, T., Finér, L., Dawud, S. M., Vesterdal, L., and Raulund-Rasmussen, K. (2015). Does species richness affect fine root biomass and production in young forest plantations? *Oecologia* 177, 581–594.

Finér, L., Domisch, T., Dawud, S. M., Raulund-Rasmussen, K., Vesterdal, L., Bouriaud, O., et al. (2017). Conifer proportion explains fine root biomass more than tree species diversity and site factors in major European forest types. *For. Ecol. Manage.* 406, 330–350.

Kaštovská, E., Straková, P., Edwards, K., Urbanová, Z., Bárta, J., Mastný, J., et al. (2018). Cotton-grass and blueberry have opposite effect on peat characteristics and nutrient transformation in peatland. *Ecosystems* 21, 443–458.

Lei, P. and Bauhus, J. (2010). Use of near-infrared reflectance spectroscopy to predict species composition in tree fine-root mixtures. *Plant Soil* 333, 93–103.

Levy, P., van Dijk, N., Gray, A., Sutton, M., Jones, M., Leeson, S., et al. (2019). Response of a peat bog vegetation community to long-term experimental addition of nitrogen. *J. Ecol.* 107, 1167–1186.

Mäkiranta, P., Laiho, R., Mehtätalo, L., Straková, P., Sormunen, J., Minkkinen, K., et al. (2018). Responses of phenology and biomass production of boreal fens to climate warming under different water-table level regimes. *Global Change Biol.* 24, 944–956.

Meinen, C. and Rauber, R. (2015). Root discrimination of closely related crop and weed species using FT MIR-ATR spectroscopy. *Front. Plant Sci.* 6, 765.

Murphy, M. T. and Moore, T. R. (2010). Linking root production to aboveground plant characteristics and water table in a temperate bog. *Plant Soil* 336, 219–231.

Nakaji, T., Noguchi, K., and Oguma, H. (2008). Classification of rhizosphere components using visible–near infrared spectral images. *Plant Soil* 310, 245–261.

Nilsson, M., Sagerfors, J., Buffam, I., Laudon, H., Eriksson, T., Grelle, A., et al. (2008). Contemporary carbon accumulation in a boreal oligotrophic minerogenic mire–a significant sink after accounting for all C-fluxes. *Global Change Biol.* 14, 2317-2332.

Picon-Cochard, C., Pilon, R., Revaillot, S., Jestin, M., and Dawson, L. (2009). Use of near-infrared reflectance spectroscopy to predict the percentage of dead versus living grass roots. *Plant Soil* 317, 309–320.

Streit, J., Meinen, C., Nelson, W. C. D., Siebrecht-Schöll, D. J., and Rauber, R. (2019). Above-and belowground biomass in a mixed cropping system with eight novel winter faba bean genotypes and winter wheat using FTIR spectroscopy for root species discrimination. *Plant Soil* 1–18.

Tong, J., Xiang, W., Lei, P., Liu, J., Tian, D., Deng, X., et al. (2016). Prediction of tree species composition in fine root mixed samples using near-infrared reflectance spectroscopy. *Plant Biosyst.* 150, 412–419.
